# Perturbation of EPHA2 and EFNA1 *trans* binding amplifies inflammatory response in airway epithelial cells

**DOI:** 10.1016/j.isci.2025.111872

**Published:** 2025-01-22

**Authors:** Ryosuke Fukuda, Shiori Beppu, Daichi Hinata, Yuka Kamada, Tsukasa Okiyoneda

**Affiliations:** 1 Department of Biomedical Sciences, School of Biological and Environmental Sciences, Kwansei Gakuin University, Hyogo 669-1330, Japan

**Keywords:** Biological sciences, Biochemistry, Cell biology

## Abstract

The interactions between EPH receptors and ephrin (EFN) ligands play a crucial role in maintaining epithelial integrity and aiding in defense against infections. However, it remains unclear how the EPH-EFN *trans*-binding changes during infections and how this alteration affects inflammatory response. Here we report that pathogen-associated molecular patterns (PAMPs) disrupt the EPHA2-EFNA1 *trans*-binding in airway epithelial cells (AECs). Mechanistically, flagellin induces the TLR5-dependent EFNA1 cleavage through the metalloproteinase ADAM9 concomitant with the activation of ligand-independent EPHA2 signaling. We found that the ablation of EPHA2 reduced the responsiveness of respiratory inflammation induced by flagellin and *Pseudomonas aeruginosa* both *in vitro* and *in vivo*. Notably, even in the absence of PAMPs, the inflammatory response in AECs was stimulated by forcibly induced EFNA1 shedding. These findings illustrate that the perturbation of the EPHA2-EFNA1 *trans*-binding acts as a sensing mechanism for infections and amplifies the inflammatory response, providing a defense mechanism for respiratory epithelia.

## Highlights

• Infectious stimuli transiently disrupt EPHA2-EFNA1 *trans*-binding in AECs
• Flagellin induces TLR5-dependent ADAM9-mediated EFNA1 cleavage in AECs
• EPHA2 ablation declines the respiratory inflammatory response to pathogens
• EPHA2-EFNA1 dissociation may function as an amplifying system for AEC inflammation

## Graphical abstract

**Figure:**
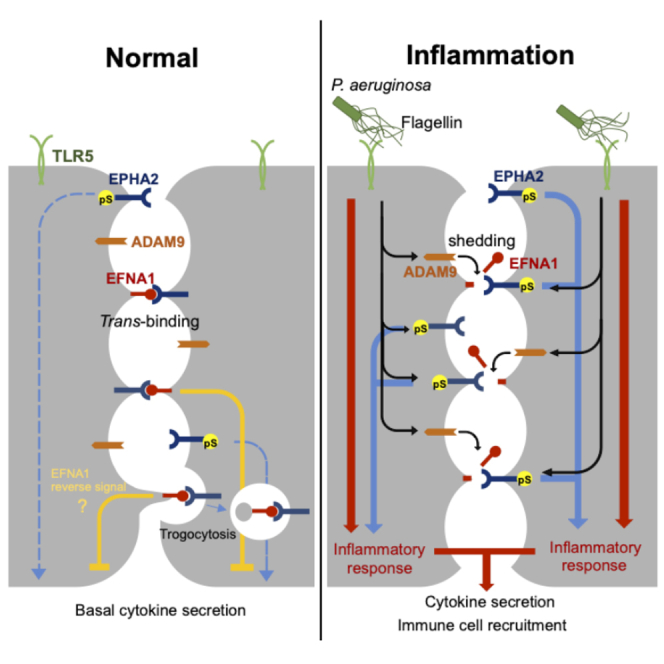

## Introduction

The AECs serve as the primary physical barrier against pathogenic infections such as bacteria and viruses and play a role in responding to infection stimuli. While chronic or excessive inflammation can lead to tissue destruction and fibrosis, inadequate inflammation can result in pathogen colonization. Thus, the inflammatory response of epithelial cells requires precise spatiotemporal regulation. Several pattern recognition receptors (PRRs) have demonstrated their role in enabling epithelial cells to identify pathogens. PRRs exhibit notable specificity for their corresponding ligands, but they also demonstrate a certain level of redundancy. This redundancy serves as a mechanism to effectively manage a wide array of pathogens.^1^^,^^2^ These innate immune responses contribute to the defense of the immature immune system during infancy and the development of acquired immunity.^3^^,^^4^

EPH receptors constitute the most extensive subgroup among receptor tyrosine kinases.^5^^,^^6^ These receptors participate in intercellular signaling and are present in cytonemes, which are filopodia extending from cells and capable of relaying signals to adjacent cells.^7^ When EPH receptors bind to their ligands EFN, they can form cell-cell contact sites through *trans*-binding. There are 14 genes of EPH receptors (EPHA1, A2, A3, A4, A5, A6, A7, A8, A10, B1, B2, B3, B4, B6) and 8 genes of EFN ligands (EFNA1, A2, A3, A4, A5, B1, B2, B3), each with different expression patterns, expression timing, and binding affinities.^6^^,^^8^ By combining various binding patterns, they contribute to maintaining tissue homeostasis and immunity.^9^^,^^10^ EPH receptors initiate distinct signaling pathways based on the presence or absence of ligand binding.^11^ For example, in the case of EPHA2, the binding of the ligand results in the phosphorylation of tyrosine residues, oligomerization, localization to signaling lysosomes, and long-term signaling.^7^ This leads to the inhibition of cell proliferation and migration through the inhibition of Akt and MAPK pathways.^11^^,^^12^ On the other hand, in the absence of a ligand, serine residues of EPHA2 such as S897 can be phosphorylated, thereby promoting cell proliferation and migration.^11^^,^^12^^,^^13^ EPHA2 can function as a receptor for β-glucan and participate in the intracellular invasion of *Candida albicans*.^14^ EPHA2 and EPHA4 have also been reported to act as entry receptors of Kaposi’s sarcoma-associated herpesvirus (KSHV).^15^^,^^16^

While EPH-EFN binding is thought to be important in maintaining the integrity of the epithelium and aiding in infection defense, it remains unclear how EPH-EFN *trans*-binding changes during inflammation and how it influences the inflammatory reactivity in the AECs. Here we report that an inflammatory signal mediated by PRRs prompts the cleavage of the EFNA1 ligand and a temporary disruption of the *trans*-binding between EPHA2 or EPHA4 and EFNA1. We show that ADAM9 is involved in the flagellin-induced EFNA1 cleavage, resulting in EPHA2 and EFNA1 dissociation. The pathogenic or forcible dissociation of the EPHA2 and EFNA1 *trans*-binding induced pro-inflammatory cytokine production in AECs. These results propose a mechanism in which the perturbation of the EPHA2/4-EFNA1 *trans*-binding amplifies inflammatory signals, thereby facilitating appropriate pathogen elimination in AECs.

## Results

### EPHA2 deficiency suppresses the respiratory inflammatory response induced by *P. aeruginosa* in mice

**Figure 1:**
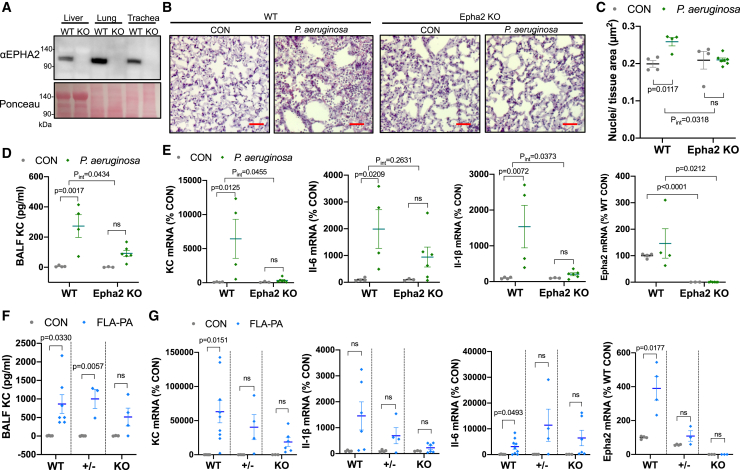
Epha2 deficiency reduces inflammatory responses induced by flagellin or *P. aeruginosa* in mouse lungs (A) EPHA2 protein expression in tissues recovered from WT and Epha2 KO mice was analyzed by WB. Ponceau staining was used as a `loading control. (B–E) WT and Epha2 KO mice were infected with 1 × 10^9^ CFU live *P. aeruginosa* via intranasal administration. The inflammatory state was evaluated in BALF and lung tissue 6 h later. (B) HE-staining image of lung tissue. The scale bar represents 10 μm. (C) Quantitative analysis of the number of cell nuclei per tissue area (μm^2^). *n* = 4, 4, 4, 6 (from left to right, two-way ANOVA, Tukey’s test). (D) ELISA of KC in BALF. *n* = 4, 4, 3, 5 (from the left lane). (two-way ANOVA, Tukey’s test). (E) RT-qPCR analysis of inflammatory cytokine and EphaA2 mRNA expression levels in lung tissue. All primer information used in this study is listed in Table 1. *n* = 4, 4, 3, 6 (from left to right, KC, Il-6, Il-1β: two-way ANOVA, Tukey’s test, Epha2: unpaired multiple *t*-test, Bonferroni-Dunn test). (F and G) WT, Epha2 +/−, and KO mice were administered 1 μg of FLA-PA via intra-tracheal injection. The respiratory inflammatory state was evaluated by using BALF and lung tissue samples collected 6 h after FLA-PA treatment. (F) ELISA of KC in BALF. *n* = 4, 7, 4, 3, 3, 4 (from left to right, unpaired *t*-test, two-tailed). (G) RT-qPCR Analysis of mRNA expression levels in lung tissue. KC and IL-6: *n* = 5, 9, 4, 4, 3, 6, Il-1β: *n* = 4, 6, 4, 4, 3, 6, Epha2: *n* = 4, 4, 4, 3, 3, 3 (from left to right, KC, Il-6, Il-1β: unpaired *t*-test, two-tailed, Epha2: unpaired multiple *t*-test, Bonferroni-Dunn test). Data are presented as mean ± SEM (C, D, E, F, G).

Infection of the airways by *Pseudomonas aeruginosa* (*P. aeruginosa*) can cause pneumonia and bronchitis, and particularly in patients with cystic fibrosis, chronic infection can lead to severe respiratory dysfunction. We initially established Epha2-knock out (KO) mice and infected with *P. aeruginosa* to investigate the function of EPHA2 in bacterial-induced airway inflammation. We then analyzed the inflammatory pathology in the respiratory system of the mouse models. Removal of Exon 6 by CRISPR-Cas9 system results in loss of amino acids that include the fibronectin type-III-2, transmembrane, and subsequent intracellular domains, leading to the loss of functional Epha2 expression (Figures 1A S1A, and S1B). Live *P. aeruginosa* was nasally administrated to WT and Epha2 KO mice and their inflammatory responses were evaluated 6 h later. While the number of infiltrating cells to the lung tissue after *P. aeruginosa* infection was significantly increased in WT mice compared to the control (CON) group, there was almost no increase observed in Epha2 KO mice (Figures 1B and 1C). In line with this observation, the levels of KC, a mouse IL-8 homolog, induced by *P. aeruginosa* in bronchoalveolar lavage fluid (BALF) from Epha2 KO mice were significantly decreased, amounting to approximately one-third of what was observed in WT mice (Figure 1D). Analysis of inflammatory marker mRNA expression using RT-qPCR revealed that the infection with *P. aeruginosa* resulted in a significant increase in the expression of KC and Il-1β in WT mice lung tissue, whereas no significant increase was observed in Epha2 KO mice (Figure 1E). On the other hand, Epha2 KO did not significantly affect IL-6 expression induction by *P. aeruginosa* (Figure 1E). Next, we investigated the involvement of EPHA2 in inflammation induced by *P. aeruginosa*-derived flagellin (FLA-PA), a major inducer of inflammatory response from *P. aeruginosa*.^17^^,^^18^ FLA-PA was administered intratracheally to WT, Epha2 heterozygous (+/−), and KO mice and we subsequently analyzed the resulting inflammatory state. The amount of KC in BALF was significantly increased by the FLA-PA treatment in WT and Epha2 +/− mice, while it was only marginally increased in Epha2 KO mice (Figure 1F). KC mRNA expression in lung tissue was significantly upregulated by FLA-PA treatment in WT mice, but this effect was attenuated in both Epha2^+/−^ and KO mice (Figure 1G). FLA-PA-induced Il-1β mRNA expression was not statistically significant but showed a similar trend to the KC results (Figure 1G). FLA-PA treatment led to a modest yet statistically significant increase in IL-6 mRNA expression in WT mice, but this effect was not suppressed in Epha2 deficient mice (Figure 1G). IL-6 expression during inflammation is more dependent on immune cells than on epithelial cells compared to IL-8 or IL-1β.^19^ Therefore, it is possible that the levels of IL-6 expression may not accurately reflect the inflammatory control function of EPHA2 in epithelial tissues, unlike in *in vitro* conditions. The expression of Epha2 decreased sequentially in the order of WT, Epha2 +/−, and KO (Figures 1E and 1G), and it was observed to increase in WT mice following FLA-PA treatment (Figure 1G). These findings unequivocally demonstrate that EPHA2 plays a pivotal role in the inflammatory response to *P. aeruginosa* infection and flagellin within the respiratory system *in vivo*.

**Table 1:** EPH and EFN plasmid list

Gene	Plasmid ID	Source
EPHA2	HsCD00516390	DNASU
EPHA2	#23926	Addgene
EPHA3	HsCD00745531	DNASU
EPHA4	HsCD00860442	DNASU
EPHA5	HsCD00860790	DNASU
EPHA6	HsCD00860443	DNASU
EPHA7	HsCD00506022	DNASU
EPHA8	HsCD00080451	DNASU
EPHA10	HsCD00860538	DNASU
EPHB1	HsCD00860535	DNASU
EPHB2	HsCD00746594	DNASU
EPHB3	HsCD00746484	DNASU
EPHB4	HsCD00829738	DNASU
EPHB6	HsCD00860137	DNASU
EFNA1	HsCD00042739	DNASU
EFNA2	HsCD00080343	DNASU
EFNA3	HsCD00040628	DNASU
EFNA4	HsCD00506902	DNASU
EFNA5	HsCD00515021	DNASU
EFNB1	HsCD00040779	DNASU
EFNB2	HsCD00352071	DNASU
EFNB3	HsCD00515523	DNASU

### EPHA2 and EFNA1 expression potentiates the *P. aeruginosa*-derived flagellinA-induced inflammatory responses in airway epithelial cells

**Figure 2:**
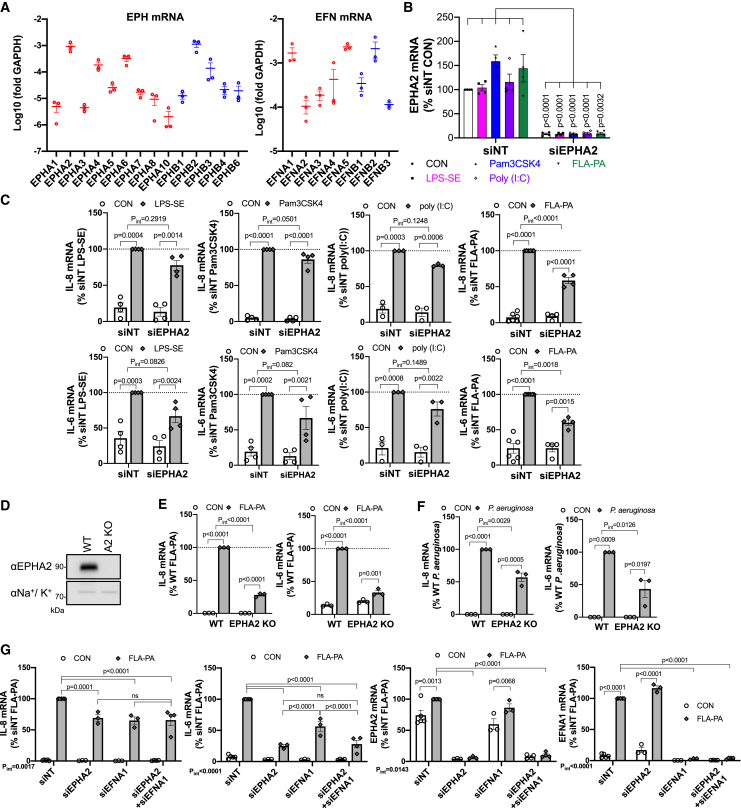
Gene silencing of EPHA2 attenuates the inflammatory response to PAMPs in AECs (A) The RT-qPCR analysis of EPH and EFN family mRNA expression in BEAS-2B cells (*n* = 3). (B and C) BEAS-2B parental cells were transfected with 20 nM EPHA2 siRNA and treated with indicated 1 μg/mL LPS-SE, 1 μg/mL PamCSK4, 1 μg/mL poly (I:C) and 100 ng/mL FLA-PA for 4 h. IL-8 and IL-6 mRNA expression was analyzed by RT-qPCR. (B) Confirmation of EPHA2 KD efficiency by siRNA (*n* = 4, *p* value: vs. siNT, unpaired multiple *t*-test, Bonferroni-Dunn test). (C) IL-8 and IL-6 mRNA expression analysis. LPS and Pam: *n* = 4, poly (I:C): *n* = 3, FLA-PA: siNT; *n* = 5, siEPHA2; *n* = 3. *p* value: vs. siNT-PAMPs, RM two-way ANOVA, Tukey’s test. (D) EPHA2 protein expression in WT and EPHA2 KO BEAS-2B cell lines. Na^+^/K^+^ ATPase was used as a loading control. (E and F) IL-8 and IL-6 mRNA expression was analyzed by RT-qPCR 4 h after treatment of 100 ng/mL FLA-PA (E) or MOI 10 live *P. aeruginosa* (F) in WT and EPHA2 KO BEAS-2B cells. *n* = 3, *p* value: vs. WT-PAMPs, RM two-way ANOVA, Tukey’s test. (G) BEAS-2B cells were transfected with 20 nM siRNA (siEPHA2, siEFNA1, or both) and treated with 100 ng/mL FLA-PA for 4 h. The expression of the inflammatory cytokines, EPHA2 and EFNA1, was analyzed using RT-qPCR. siNT; *n* = 5, siEPHA2; *n* = 3, siEFNA1; *n* = 3, siEPHA2+EFNA1; *n* = 4, RM two-way ANOVA, Tukey’s test. Data are presented as mean ± SEM (A, B, C, E, F, G).

To ascertain the necessity of EPHA2 in the inflammatory responses triggered by different PAMPs, we performed gene silencing by siRNA knockdown (KD) and EPHA2 KO using the CRISPR/Cas9 system (Figure S2A). We selected the bronchial epithelial cell line BEAS-2B for our analysis based on its responsiveness to PAMPs and its high basal expression levels of EPHA2 and EFNA1^18^ (Figure 2A). The inflammatory responses to PAMPs such as *Salmonella enterica* (SE)-derived lipopolysaccharide (LPS-SE), Pam3CSK4, poly(I:C), and FLA-PA were measured by assessing the induction of inflammatory cytokine mRNA 4 h after stimulation using RT-qPCR analysis. The KD efficiency was also confirmed by RT-qPCR (Figure 2B). The IL-8 induction triggered by LPS-SE, Pam3CSK4, and Poly(I:C) tended to be suppressed by EPHA2 KD, although the suppression was not statistically significant (Figure 2C). In contrast, the KD of EPHA2 resulted in the significant reductions in the inflammatory responses elicited by FLA-PA (Figure 2C).

The FLA-PA-induced inflammatory response upon EPHA2 KO BEAS-2B cell was also examined. We confirmed EPHA2 KO by genome sequencing and Western blotting (Figures S2A and 2D). The EPHA2 KO reduced the FLA-PA-induced IL-8 and IL-6 expression consistent with the observations in the KD cells (Figure 2E). Similar results were obtained using live *P. aeruginosa* as the EPHA2 KO reduced the expression of inflammatory cytokines IL-8 and IL-6 in the infected BEAS-2B cells (Figure 2F).

An analysis of the timeline revealed that the peak of IL-8 expression induced by FLA-PA occurred at 4-h, and the consistent suppression of the inflammatory response by EPHA2 KO was observed at all time points (Figure S2B). This result suggests that the pathway involving EPHA2 may serve to amplify the inflammatory response induced by the PAMPs in AECs.

We next examined the role of EFNA1 in the PAMPs-induced inflammatory response. RT-qPCR analysis showed that such as EPHA2 KD, EFNA1 KD reduced the induction of IL-8 and IL-6 upon FLA-PA treatment in BEAS-2B cells (Figure 2G). However, there was no additive effect of the double KD, suggesting that EPHA2 and EFNA1 contribute to the FLA-PA-induced inflammation through the same pathway (Figure 2G). Furthermore, we observed a substantial approximately 10-fold increase in EFNA1 due to the FLA-PA stimulation, whereas EPHA2 exhibited only a slight approximately 1.3-fold increase (Figure 2G). These findings suggest that the EPHA2-EFNA1 axis contributes to the inflammatory signaling activated by PAMPs such as FLA-PA and living *P. aeruginosa* infection.

### Establishment of the EPH-ephrin *trans*-binding measurement in airway epithelial cells

**Figure 3:**
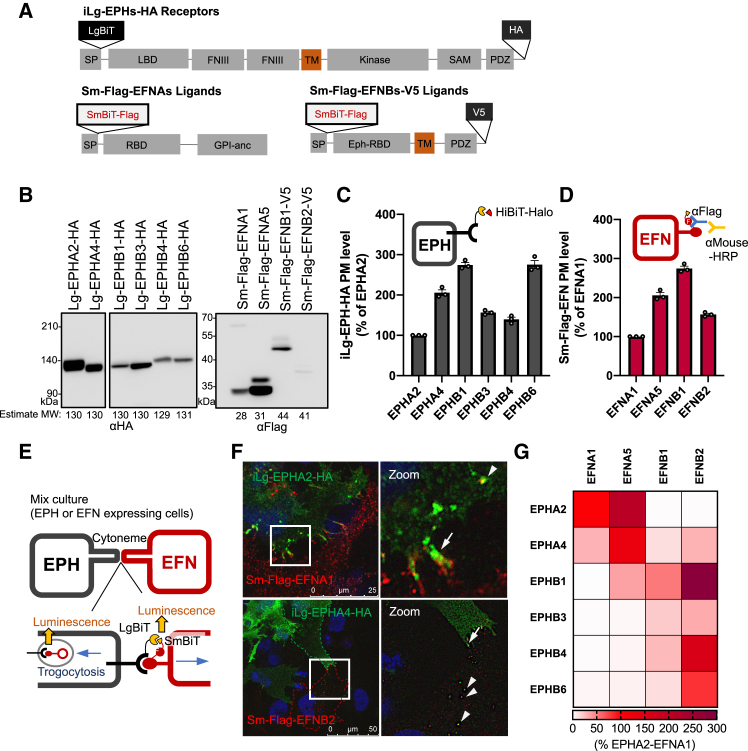
Establishment of EPH-EFN *trans*-binding real-time measurement in live AECs (A) Schematic of established iLgBiT-EPH-HA and SmBiT-Flag-EFN(-V5) constructs. Original plasmid information of EPH and EFN genes used in this study is listed in Table 2. SP: signal peptide, LBD: ligand binding domain, FNIII: fibronectin-type III, TM: transmembrane domain, SAM: sterileαmotif, PDZ: PDZ-binding, RBD: receptor binding domain, GPI-anc: GPI anchor propeptide region. (B) Western blotting analysis of iLg-EPH-HA and Sm-Flag-EFN protein expression in BEAS-2B cells with stable expression. iLg-EPH-HA expression cells were treated with 1 μg/mL Dox for 72 h to induce their expression. The exogenous EPHs and EFNs were detected by anti-HA antibody and anti-Flag antibody, respectively. The expected molecular weight (MW: kDa) were shown below each band. (C) Plasma membrane expression analysis of iLg-EPH-HA in BEAS-2B cells by using HiBiT-Halo protein. iLg-EPH-HA expression was induced by treatment of Dox (A2, A4: 50 ng/mL, B1: 500 ng/mL, B3: 150 ng/mL, B4, B6: 1000 ng/mL) for 48 h (*n* = 3). (D) PM expression of Sm-Flag-EFN in BEAS-2B cells was measured by cell-surface ELISA using anti-Flag antibody and HRP-conjugated anti-mouse antibody (*n* = 3). (E) Schematic of EPH-EFN *trans*-binding measurement using iLg-EPH-HA-expressing cells (EPH, black) and Sm-Flag-EFN-expressing cells (EFN, red) co-culturing system. EPH-EFN binding is formed in cytonemes contact site between EPH or EFN expressing cells, and the signal of complemented NanoLuc reflects the amount of EPH-EFN interaction. Membrane-permeable substrate enables to detect EPH-EFN interactions inside cells. (F) Immunostaining of the cell-cell contact site during the co-culturing of iLg-EPHA2-HA and Sm-Flag-EFNA1 or iLg-EPHA4-HA and Sm-Flag-EFNB2-V5 expressing cells. The iLg-EPH-HA were labeled in green and the Sm-Flag-EFN were labeled in red. Outline of iLg-EPHA4-HA expressing cell (Green) or Sm-Flag-EFNB2-V5 (Red) expressing cells was shown in broken line. The right panel shows an enlarged image of the white box area of left panel, and white arrowhead shows co-localization. (G) The quantitative results of EPH-EFN *trans*-binding measurement between iLg-EPH and Sm-Flag-EFN in BEAS-2B cells were displayed as a heatmap, with the *trans*-binding amount relative to EPHA2-EFNA1 pair (*n* = 3). Data are presented as mean ± SEM (C, D).

To elucidate the function of the EPHA2-EFNA1 binding during pathogen infection, we investigated whether pathogen stimuli affect the EPHA2-EFNA1 binding in BEAS-2B cells. To this end, we developed an assay system to easily measure the EPH-EFN *trans-*binding in real-time on live cells. We exploited the NanoBiT system, a protein interaction analysis using proximity-dependent NanoLuc reconstitution,^20^ for the measurement of the EPH-EFN *trans-*binding. We selected EPHA2, A4, EPHB1, B3, B4, B6, EFNA1, A5, EFNB1 and B2 which were confirmed to be expressed in BEAS-2B cells as EPH-EFN pairs (Figure 2A). We constructed lentiviral vectors that allow doxycycline (Dox)-inducible (i) expressions of LgBiT (Lg)-EPH-HA in which Lg was fused in the upstream of the ligand binding domain (LBD) and HA-tag at the C-terminus of EPHA2, 4 or EPHB1, 3, 4, 6 (Figure 3A). We also generated a lentiviral vector that enables the constitutive expression of EFNA1, A5, B1, B2 fused with SmBiT and FLAG tag (Sm-Flag) in the upstream of the receptor binding domain (RBD) (Figure 3A). The V5-tag was also added to the C-terminal intracellular region of EFNB1, 2 (Figure 3A). The localization of iLg-EPH-HA and Sm-Flag-EFN in BEAS-2B cells was confirmed, and the membrane expression of the exogenous EPHs and EFNs was observed in all constructs (Figure S3A). Protein expression levels of each construct were also confirmed by Western blotting (WB) (Figure 3B). The plasma membrane (PM) expression levels of iLg-EPH-HA or Sm-Flag-EFN in established cell lines were confirmed by cell-based ELISA since the amount of EPH-EFN *trans*-binding depends on the PM expression levels of each EPHs and EFNs (Figures 3C and 3D). The PM expression of EFNB2 was higher than that of EFNA1 despite its lower protein level, indicating that EFNB2 could be efficiently translocated to the PM (Figures 3B and 3D).

**Table 2:** Q-RT-PCR primer list

Gene	Fw (5′ to 3′)	Rv (5′ to 3′)
Gapdh (mouse)	CCTGGAGAAACCTGCCAAGTATG	GGTCCTCAGTGTAGCCCAAGATG
Il-6 (mouse)	GAGGATACCACTCCCAACAGACC	AAGTGCATCATCGTTGTTCATACA
KC (mouse)	ACCCAAACCGAAGTCATAGC	TCTCCGTTACTTGGGGACAC
Tnf-α (mouse)	CATCTTCTCAAAATTCGAGTGACAA	TGGGAGTAGACAAGGTACAACCC
Il-1β (mouse)	GCTGAAAGCTCTCCACCTCAATG	TGTCGTTGCTTGGTTCTCCTTG
Epha2 (mouse)	GTGTGACAGTCAGTGACCTG	CACGGCTCTGCTGTGACAC
GAPDH	CATGAGAAGTATGACAACAGCCT	AGTCCTTCCACGATACCAAAGT
EPHA1	GTGGACACTGTCATAGGAGAAGG	GGTCTTAATGGCCACAGTCTTG
EPHA2	TGTGCCAGGCAGGCTACG	CTCCAAGCAGGGGCTCTCA
EPHA3	GATGTTGGTGCTTGTGTTGC	GTGTCTGGAAACATAGCCAGATT
EPHA4	ACCAAGCAGTGCGAGAGTTTGCC	TCTCTCTTGCCAGGCACTTTGAG
EPHA5	ACTTGATCTTGGTGACCGTGT	ACCAGAGCAATGCAAGCAC
EPHA6	TGAAGACCCATCCCTAGCAG	AGACTTCTCCAAATTCACCTGC
EPHA7	TGGGAAGAAATTAGTGGTTTGG	GTTAGTCCGCAGCCAGTTGT
EPHA8	GACACACCCAAGATGTACTGCA	GTAGAAGCCCAGCTCACAGG
EPHA10	CCAAGTGTGCCCTGACTACCTGTC	GTTCAGCCAAAGAGATGCCTAGGCTCAC
EPHB1	GCACATCTCTGGTGATTGCTC	ACGCTGTTCTCAGGCTCATAG
EPHB2	GAAGGAGCTCAGTGAGTACAACG	GCACCTGGAAGACATAGATGG
EPHB3	GGCCATAGCCTATCGGAAGT	TCCCAGTAGGGTCGCTCTC
EPHB4	GCCATTGAACAGGACTACCG	TTCCGGATCATCTTGTCCA
EPHB6	GAGCAGGAGGTACTAAATGCAA	CCAGCTGGTCAAAATGAGG
EFNA1	ATCGCCACACCGTCTTCTG	CACGTAGTCATTCAGCTGCACAT
EFNA2	TGGAGGTGAGCATCAATGAC	CCGTTGACCATGTACAGCAC
EFNA3	GGATGAAGGTGTTCGTCTGC	TTCTCTCCCTCAAAGTCTTCCA
EFNA4	CTCCAGGTGTCTGTCTGCTG	AGTAATAGCAAGAGACAGAG
EFNA5	TCTCCAAATGGACCGCTGAAGTTC	AGCTTTAGACAGGACCTTCTTCC
EFNB1	TGAAGGTTGGGCAAGATCC	GGTTCACAGTCTCATGCTTGC
EFNB2	GGAGGAGACACAGGAAGCAC	CGTAGTGAGGGCAGAAGACG
EFNB3	CTTCACCATCAAGTTCCAGGA	ATGCCTCTGGTTAGGCACAC
IL-8	TCCTGATTTCTGCAAGCTCTG	GTCCACTCTCAATCACTCTCAG
IL-6	GCACTGGCAGAAAACAACCT	CAGGGGTGGTTATTGCATCT
ADAM9	CCTCGGGGACCCTTCGTGT	ATCCCATAACTCGCATTCTCTAAA
ADAM12	CAGCCAAGCCTGCACTTAG	AGTGAGCCGAGTTGTTCTGG
ADAM15	AGCCTCAAAAAGGTGCTTCA	CCCTGGTAGCAGCAGTTCTC
MMP14	CCTGATAAGCCCAAAAACC	CTTCCTCTCATAGGCAGTGTT
MMP15	CATGCGTTCCGCCCAGAT	CCGCAGGTTGGCTTTCAC

EPH-EFN *trans*-binding can occur at contact sites between the cytonemes of adjacent cells, resulting in the EPH-EFN complex being endocytosed into the cell by trogocytosis.^7^^,^^21^ To measure the amount of EPH-EFN *trans*-binding, iLg-EPH-HA expressing cells and Sm-Flag-EFN expressing cells were mixed and co-cultured, resulting in the EPH-EFN *trans*-binding at the cell-cell contact site (Figure 3E). In this experimental system, exogenous EPH and EFN were transduced into separate cell clones, respectively, and then both cell types were co-cultured to minimize the likelihood of *cis-*interactions. Immunofluorescence staining revealed the co-localization of exogenous EPHA2-EFNA1 and EPHA4-EFNB2 pairs in the cytonemes of the cell adhesion site (Figure 3F), both of which have been previously reported to interact.^8^ Interestingly, the EPHA2-EFNA1 binding in intracellular vesicles was observed in the iLg-EPHA2-HA expressing cells, whereas the intracellular EPHA4-EFNB2 co-localization was observed in the vesicles in the Sm-Flag-EFNB2-V5 expressing cells (Figure 3F, arrowhead). This result suggests that the exogenously expressed EPH-EFN forms *trans*-binding at the cell-cell adhesion site and the EPH-EFN complexes are *trans*-endocytosed to endosomes. Next, iLg-EPH-HA and Sm-Flag-EFN expressing cells were co-cultured with different combinations, and the signal generated by the NanoLuc reconstruction was measured and quantified as the amount of EPH-EFN *trans*-binding (Figures 3G and S3B). The data showed that EPHA2 interacted with EFNA1, EFNA5, EPHA4 with EFNA1, EFNA5, EFNB1, EFNB2, EPHB1 with EFNA5, EFNB1, EFNB2 and EPHB3, 4, 6 with EFNB1, EFNB2 (Figures 3G and S3B). The co-localization of EPH-EFN pairs found to bind by the NanoBiT assay was confirmed by immunofluorescence staining (Figure S3C). The EPH-EFN binding patterns observed in this analysis were consistent with previous *in vitro* studies,^8^^,^^22^ with the exception of EPHA4-EFNB1 and EPHB1-EFNA5, which were uniquely detected in our analysis. The functional significance of the observed EPHA4-EFNB1 and EPHB1-EFNA5 *trans-*binding remains uncertain. Inconsistent with the previous study,^8^ we were unable to detect the EPHB1-EFNA1 *trans*-binding (Figure 3G). In this experimental system, LgBiT or SmBiT tags were inserted just below the signal sequences of EPH and EFN, potentially affecting the conformation of the binding site and hindering the replication of physiological EPH-EFN binding. However, this method can conveniently measure the amount of specific EPH-EFN *trans*-binding between living cells, at least for certain types of EPH-EFN *trans*-binding, as it is consistent with previous *in vitro* studies.^8^^,^^22^

### Quantitative analysis of pathogenic factors effect on EPH-ephrin *trans-*binding

**Figure 4:**
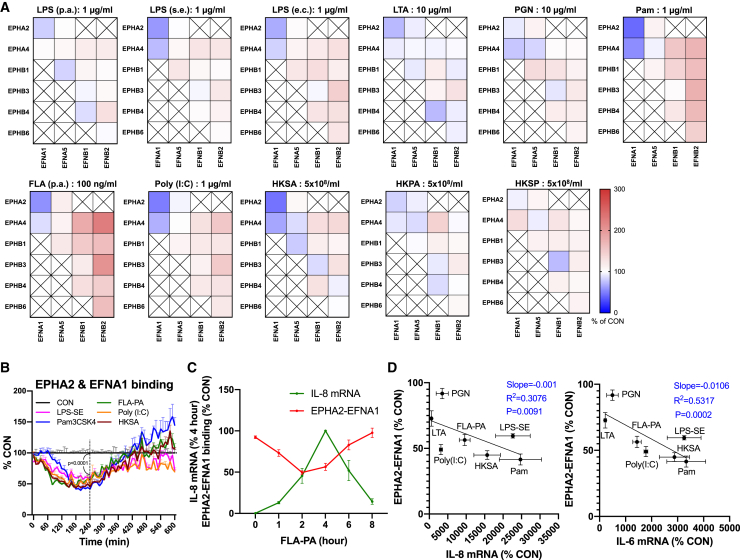
The impact of pathogen components on the *trans*-binding of EPH and EFN (A) Real-time EPH-EFN *trans*-binding measurement was performed during the treatment of PAMPs and heat-killed bacteria. We presented relative changes in EPH-EFN binding compared to the control (CON) at the 4-h after stimulation in a heatmap. All the experimental results over the entire time course are presented in Figure S4 (*n* = 3). (B) Time course analysis of the changes in EPHA2-EFNA1 *trans*-binding following treatment with specified pathogen molecules. We presented the statistical significance of the results at the 4-h after treatment (*n* = 3). RM one-way ANOVA, Dunnett’s test. (C) Comparison of the time course of inflammatory response and the changes in EPHA2-EFNA1 *trans*-binding during treatment with 100 ng/mL FLA-PA in BEAS-2B cell. The inflammatory response was assessed by analyzing IL-8 mRNA level by RT-qPCR (*n* = 3). (D) The correlation between EPHA2-EFNA1 *trans*-binding (Figure 4A) and IL-8 or IL-6 mRNA induction (Figure S4B) in BEAS-2B cells 4 h after each stimulation. Data are presented as mean ± SEM (B (+SEM only), C, D).

Using the established live-cell EPH-EFN *trans-*binding measurement, we investigated the effect of pathogenic factors on EPH-EFN binding. Pathogens used were heat-killed (HK) *Staphylococcus aureus* (SA), *P. aeruginosa* (PA), and *Staphylococcus pneumonia* (SP), and pathogenic factors including LPS-PA, LPS-SE, LPS-*Escherichia coli* (EC), lipoteichoic acid (LTA), peptidoglycan (PGN), Pam3CSK4 (Pam), FLA-PA, and poly (I:C). LPS from several pathogens were used to investigate whether variations in LPS structures result in different effects. The EPH-EFN *trans-*binding was measured at 10-min intervals for 10 h after pathogens treatment, and the relative change compared with solvent treated CON group at all time points was continuously monitored (Figure S4A). We showed the results at 4 h after treatment when the inflammatory response was maximized in AECs under FLA-PA treatment (Figures 4A and 4C). Interestingly, LPS, Pam, FLA-PA, Poly (I:C) and HKSA treatment transiently induced 40–60% decrease in EPHA2-EFNA1 *trans-*binding at 2 to 4 h after stimulation (Figures 4A, 4B, and S4A). In addition to the EPHA2-EFNA1, the EPHA4-EFNA1 binding was also decreased by the inflammatory stimuli (Figure 4A). The analysis of the timeline revealed that the peak of EPHA2-EFNA1 dissociation occurred before the peak of inflammatory cytokine induction during FLA-PA treatment (Figure 4C). This data suggests that the disruption of EPHA2-EFNA1 *trans*-binding in response to inflammatory stimulation is not merely an outcome of inflammation; rather, it appears to be a regulatory factor of the inflammatory response. We observed a low to moderate correlation between the PAMPs-induced IL-8 (R^2^ > 0.3) and IL-6 (R^2^ > 0.5) expression and the reduction in the EPHA2-EFNA1 *trans*-binding caused by PAMPs (Figures 4D and S4B). These results imply that the extent of EPHA2-EFNA1 dissociation during inflammatory stimulation might determine the magnitude of the inflammatory response in AECs. These findings imply that certain PAMPs, such as LPS, Pam, FLA-PA, and Poly (I:C), specifically diminish the *trans*-binding between EPHA2 or EPHA4 and EFNA1. This reduction in *trans*-binding could potentially initiate inflammatory responses in AECs.

### *P. aeruginosa*-derived flagellin triggers the removal of EFNA1 from the Plasma membrane in a TLR5-dependent manner

**Figure 5:**
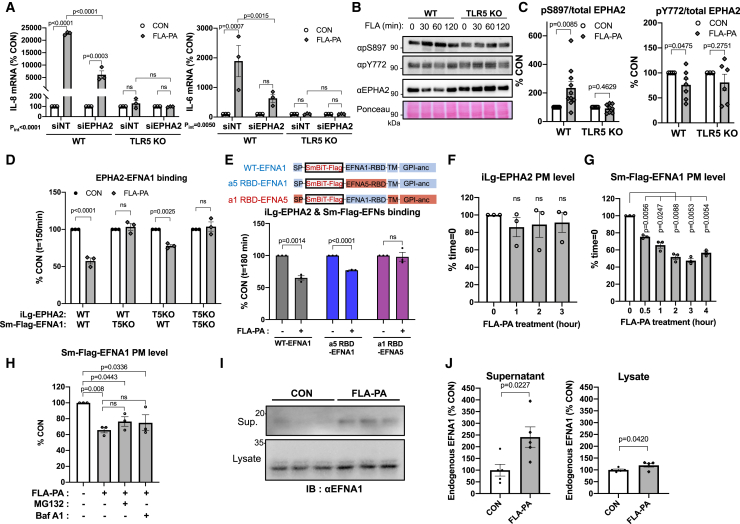
FLA-PA induces TLR5 dependent EFNA1 release from the PM in AECs (A) WT or TLR5 KO BEAS-2B cells were transfected with 20 nM EPHA2 siRNA and the FLA-PA induced inflammatory response was analyzed by RT-qPCR. *n* = 3, RM two-way ANOVA, Tukey’s test. (B) WT or TLR5 KO BEAS-2B cells were treated with 100 ng/mL FLA-PA for 0 to 120 min, and the total EPHA2, phospho-S897 (pS897) and phosphor-Y772 (pY772) EPHA2 levels were analyzed by WB. Ponceau staining was used as loading control. (C) The ratio of pS897 or pY772/total EPHA2 at 1 h after FLA-PA treatment was calculated from the intensity of the WB result. *n* = 10, 10, 8, 8 (pS897, from left to right) and *n* = 7, 7, 6, 6 (pY772, from left to right), unpaired *t-*test, two-tailed. (D) Analysis of whether the TLR5-dependent dissociation of EPHA2-EFNA1 by FLA-PA results in an effect on EPHA2 or EFNA1. WT or TLR5 KO BEAS-2B cells expressing iLg-EPHA2-HA or Sm-Flag-EFNA1 were co-cultured in indicated combinations. The EPHA2-EFNA1 *trans*-binding relative to CON was quantified at 150 min after 100 ng/mL FLA-PA treatment. *n* = 3, unpaired multiple *t*-test, Bonferroni-Dunn test. (E) The analysis of EFN-RBD dependency in FLA-PA induced EPHA2-EFNA1 dissociation. BEAS-2B cells expressing Sm-Flag fused WT-EFNA1, a5 RBD-EFNA1 or a1 RBD-EFNA5 were co-cultured with iLg-EPHA2-HA-expressing cells. The EPHA2 and WT-EFNA1, a5 RBD-EFNA1 or a1 RBD-EFNA5 *trans*-binding was measured during 100 ng/mL FLA-PA treatment. The interaction ratio relative to CON was quantified at 180 min after FLA-PA-treatment. *n* = 3, unpaired multiple *t*-test, Bonferroni-Dunn test. (F) The FLA-PA impact on EPHA2 p.m. level. iLg-EPHA2-HA expressing BEAS-2B cell were treated with 100 ng/mL FLA-PA for 0–3 h and PM expression level of iLg-EPHA2-HA was analyzed by using HiBiT-Halo protein. *n* = 3, RM one-way ANOVA, Dunnett’s test. (G) The FLA-PA impact on EFNA1 p.m. level. SmBiT-Flag-EFNA1 p.m. levels in BEAS-2B cells under 100 ng/mL FLA-PA treatment for 0–4 h was analyzed by cell surface ELISA with anti-Flag antibodies. *n* = 3, RM one-way ANOVA, Dunnett’s test. (H) Proteasomal or lysosomal degradation inhibitor effect on FLA-PA induced EFNA1 loss from PM. Sm-Flag-EFNA1 expressing BEAS-2B cells were treated with 10 μM MG132 or 1 μM Bafilomycin A1 (Baf A1) for 2 h before and during treatment with 100 ng/mL FLA-PA. PM level of Sm-Flag-EFNA1 was measured at 1 h after FLA-PA treatment by cell surface ELISA and shown as relative to CON. *n* = 3, two-way ANOVA, Tukey’s test. (I and J) FLA-PA effect on endogenous EFNA1 protein level. BEAS-2B parental cells were treated with 100 ng/mL FLA-PA for 4 h, and the endogenous EFNA1 levels in the supernatant (Sup.) and cell lysate (Lysate) were immunoblotted (IB) by using anti-EFNA1 antibody. (J) The intensity of EFNA1 band was quantified. *n* = 4, unpaired *t-*test. Data are presented as mean ± SEM (A, C, D, E, F, G, H, J).

Since FLA is recognized by TLR5 and induces IL-8 production through the NF-κB pathway as well as IL-18 production through NLRC4-mediated inflammasome activation,^23^ we investigated whether the EPHA2-mediated inflammatory response is contingent upon the TLR5 signaling pathway. To this end, we generated TLR5 KO BEAS-2B cells (Figure S2A). As expected, the expression of IL-8 and IL-6 induced by FLA-PA was completely suppressed in the TLR5 KO cells (Figure 5A). Although EPHA2 KD led to a significant decrease in FLA-PA-induced IL-8 and IL-6 expression in the parental cells, this effect was not observed in the TLR5 KO cells (Figure 5A). These findings suggest that the impact of EPHA2 on the FLA-PA-induced inflammatory response is contingent on TLR5 and that EPHA2 seems to operate downstream of the TLR5 signaling pathway.

It has been reported that the phosphorylation of the serine 897 (S897) residue takes place in EPHA2 in the absence of ligand binding, leading to ligand-independent signaling and phosphorylation of tyrosine residues such as Y772 occurs ligand dependent manner.^11^ The phosphorylation of serine residues in EPHA2 triggers the activation of signaling molecules associated with inflammatory responses such as Akt and STAT3.^24^ The Western blot analysis revealed that FLA-PA treatment led to an elevation in the phosphorylation of EPHA2 at S897 and reduction in the phosphorylation at Y772, reaching its peak in WT cells 1 h after treatment but the effect was reduced in TLR5 KO cells (Figures 5B and 5C). Thus, following FLA-PA treatment, the reduction in *trans-*binding between EPHA2 and EFNA1 coincides with EPHA2 S897 phosphorylation, and this reduction in *trans-*binding may promote the ligand-independent serine phosphorylation.

Next, we investigated whether the TLR5 signaling pathway affects the EPHA2-EFNA1 *trans-*binding itself. To accomplish this, we established WT and TLR5 KO cells expressing iLg-EPHA2-HA or Sm-Flag-EFNA1, and these cells were co-cultured in various combinations to assess the *trans*-binding interaction. FLA-PA caused a roughly 50% reduction in the EPHA2-EFNA1 *trans*-binding within the WT cell pair. However, this impact was nullified in the TLR5 KO cell pair and when Sm-Flag-EFNA1 was expressed in TLR5 KO cells (Figures 5D and S5A). These findings suggest that the TLR5 signaling triggered by FLA-PA reduces the strength of the EPHA2-EFNA1 *trans*-binding by exerting an effect on EFNA1 rather than EPHA2 (Figures 5D and S5A).

To elucidate how FLA-PA affects the function of EFNA1, we aimed to identify the specific region of EFNA1 responsible for the dissociation of EPHA2-EFNA1 induced by FLA-PA. To achieve this, we generated chimeric proteins wherein the RBD domains of EFNA1 and EFNA5 were swapped with each other. EFNA5 belongs to the same EFNA subfamily as EFNA1, but unlike EFNA1, its *trans*-binding with EPHA2 was not reduced by PAMPs such as FLA-PA (Figure 5E). We established BEAS-2B cells expressing Sm-Flag-a5 RBD-EFNA1 (EFNA1 with EFNA5 RBD) or Sm-Flag-a1 RBD-EFNA5 (EFNA5 with EFNA1 RBD). These cells were then co-cultured with cells expressing iLg-EPHA2-HA. This setup was designed to investigate whether the RBD domain of EFNA1 influences FLA-PA-induced dissociation of EPHA2-EFNA1 binding. If FLA-PA indeed targets the a1-RBD, the dissociation effect should be nullified when using a5 RBD-EFNA1. Nevertheless, FLA-PA treatment diminished the interaction between EPHA2 and a5 RBD-EFNA1, similar to its impact on WT-EFNA1 (Figures 5E and S5B). In contrast, FLA-PA did not trigger the dissociation of the interaction between EPHA2 and a1 RBD-EFNA5 (Figures 5E and S5B). These findings suggest that FLA-PA induces the dissociation of EPHA2-EFNA1 not by impeding the interaction between LBD and RBD but rather by affecting a region other than the RBD of EFNA1.

Next, we examined the temporal changes in the PM expression levels of iLg-EPHA2-HA and Sm-Flag-EFNA1 during FLA-PA treatment. The PM expression level of iLg-EPHA2-HA in BEAS-2B was not affected by FLA-PA treatment (Figure 5F). In contrast, the PM expression levels of Sm-Flag-EFNA1 decreased immediately after FLA-PA treatment, reaching their lowest level after 3 h (Figure 5G). This temporal progression aligned with the decrease in EPHA2-EFNA1 *trans*-binding induced by FLA-PA, as observed in the *trans*-binding analysis (Figure 4B).

To investigate whether the decrease in EFNA1 p.m. expression resulted from protein degradation, we employed the proteasome inhibitor MG132 and the lysosome inhibitor Bafilomycin A1 (Baf A1), which are central to the degradation pathway of membrane proteins. The data showed that even when the degradation pathway was inhibited, FLA-PA still decreased the EFNA1 p.m. expression, indicating that EFNA1 was removed from the PM through mechanisms other than degradation (Figure 5H). Next, we assessed the quantity of endogenous EFNA1 in the cells and in the cell culture medium following 4-h of FLA-PA treatment. The WB analysis showed that the amount of EFNA1 in the medium was increased by about 2.5 times in FLA-PA treatment compared to CON whereas intracellular EFNA1 was increased only by about 1.2 times (Figures 5I and 5J). This result suggests that FLA-PA promotes the release of EFNA1 into the extracellular space. Taking together, our results propose that the TLR5 signaling prompted by FLA-PA triggers the release of EFNA1 from the PM, thereby reducing the EPHA2-EFNA1 *trans*-binding.

### A metalloproteinase-9 induces the shedding of EFNA1 depending on the TLR5 signaling pathway

**Figure 6:**
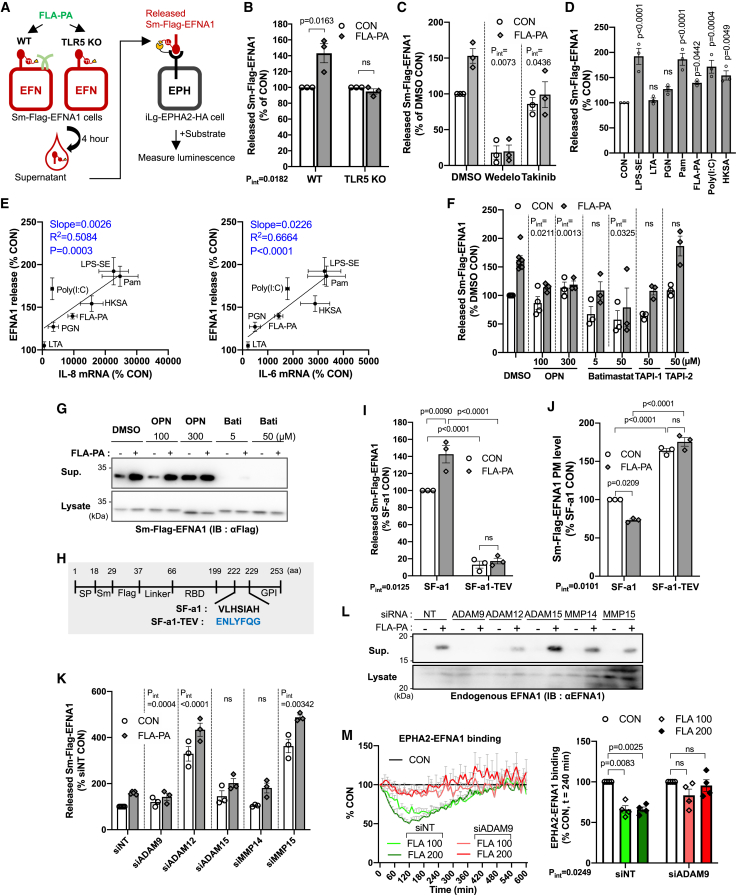
EFNA1 is cleaved by ADAM9 in response to flagellin-TLR5 signaling (A and B) Schema and result of the released Sm-Flag-EFNA1 measurement. WT or TLR5 KO BEAS-2B cells expressing Sm-Flag-EFNA1 were treated with 100 ng/mL FLA-PA for 4 h, and the supernatant was collected. Then mixture of the collected supernatant containing released Sm-Flag-EFNA1 and NanoBiT substrate were added to iLg-EPHA2-HA expressing cells. The amount of Sm-Flag-EFNA1 in the supernatant was analyzed based on NanoLuc luminescence formed between Sm-Flag-EFNA1 in the supernatant and iLg-EPHA2-HA on the cell surface. *n* = 3, RM two-way ANOVA, Sidak’s test. (C) The effect of NF-κB inhibitors on FLA-PA-induced EFNA1 release. Sm-Flag-EFNA1 expressing cells were pre-treated with 50 μM Wedelolactone or 10 μM Takinib for 2 h, and then co-treated with 100 ng/mL FLA-PA for 4 h. The supernatant was collected and released Sm-Flag-EFNA1 was measured. *n* = 3, RM two-way ANOVA, Sidak’s test. (D) The release of EFNA1 in response to various pathogenic stimuli. The measurement of released Sm-Flag-EFNA1 was conducted 4 h after treating with the same concentrations of pathogen components as depicted in Figure 2*n* = 3, RM one-way ANOVA, Dunnett’s test. (E) Correlation analysis between the Sm-Flag-EFNA1 release (Figure 6D) and the inflammatory cytokine mRNA induction (Figure S4B) after 4-h FLA-PA treatment, relative to each control (CON). *n* = 3. (F and G) The effect of MMP/ADAM inhibitors on FLA-PA-induced EFNA1 release. Sm-Flag-EFNA1 expressing cells were pre-treated with indicated concentration of MMP/ADAM inhibitors for 2 h, and then co-treated with 100 ng/mL FLA-PA for 4 h. (F) The supernatant was collected and used for released Sm-Flag-EFNA1 measurement. DMSO: *n* = 7, OPN 100 μM: *n* = 4, Other groups: *n* = 3, RM two-way ANOVA, Dunnett’s and Sidak’s test. (G) The amount of Sm-Flag-EFNA1 in the supernatant (Sup.) and cell lysate (Lysate) were analyzed by WB by using anti-Flag antibody. (H) Sm-Flag-EFNA1-TEV (SF-a1-TEV) was generated by replacing the metalloprotease recognition sequence of Sm-Flag-EFNA1 (SF-a1) with the TEV protease recognition sequence (Blue). (I and J) The metalloprotease recognition site is crucial for FLA-PA-induced EFNA1 release. BEAS-2B cells expressing SF-a1 and SF-a1-TEV were exposed to FLA-PA for 4 h. The quantities of SF-a1 or SF-a1-TEV released into the supernatant (I) or present on the cell surface (J) were determined using released SF-a1 measurement and cell surface ELISA methods. *n* = 3, RM two-way ANOVA, Sidak’s test. (K) The impact of MMPs/ADAMs KD on FLA-PA-induced exogenous EFNA1 release. BEAS-2B cells expressing Sm-Flag-EFNA1 were transfected with 20 nM siRNA targeting ADAMs or MMPs, and the amount of released Sm-Flag-EFNA1 was measured 4 h after FLA-PA treatment. siNT: *n* = 5, Other groups: *n* = 3, RM two-way ANOVA, Dunnett’s and Sidak’s test. (L) The impact of MMPs/ADAMs KD on FLA-PA-induced endogenous EFNA1 release. BEAS-2B cells were transfected with 20 nM siRNA targeting ADAMs or MMPs, and the amount of EFNA1 in the supernatant (Sup.) and cell lysate (Lysate) 4 h after 100 ng/ml FLA-PA treatment were analyzed by WB. (M) The effect of an ADAM9 KD on FLA-PA-induced EPHA2-EFNA1 dissociation. ADAM9 KD was performed on co-cultured iLg-EPHA2-HA and Sm-Flag-EFNA1 cells, and EPHA2-EFNA1 *trans*-binding was monitored during FLA-PA treatments at 100 or 200 ng/mL. The graph on the left shows the time-course changes in EPHA2-EFNA1 binding, while the graph on the right presents the statistical analysis of the results obtained 4 h after FLA-PA stimulation. *n* = 3, RM two-way ANOVA, Dunnett’s test. Data are presented as mean ± SEM (B, C, D, E, F, I, J, K, M (+SEM only)).

To investigate the mechanism of EFNA1 extracellular release by FLA-PA, we developed an assay measuring the released Sm-Flag-EFNA1 using NanoBiT technology. The cell culture media of WT or TLR5 KO BEAS-2B cells expressing Sm-Flag-EFNA1 were collected at 4 h after FLA-PA treatment. The collected media were added to the iLg-EPHA2-HA-expressing BEAS-2B cells with a NanoLuc substrate, and the luminescence generated by the interaction of Sm-Flag-EFNA1 and iLg-EPHA2-HA was quantified as the amount of released Sm-Flag-EFNA1 in the medium (Figure 6A). In the WT cells, FLA-PA increased the released Sm-Flag-EFNA1 by about 1.5 times, while no increase was observed in TLR5 KO cells (Figure 6B). Additionally, treatment with Wedeloractone or Takinib, inhibitors of NF-κB downstream of TLR5 signaling, suppressed the FLA-PA-induced Sm-Flag-EFNA1 release (Figure 6C). In addition to FLA-PA, inflammatory stimuli such as LPS-SE, Pam, Poly(I:C), and HKSA, which inhibit EPHA2-EFNA1 binding (Figures 4A and 4B), induced the release of SF-a1 into the extracellular space (Figure 6D). Furthermore, the amount of released SF-a1 was well correlated with the expression of inflammatory cytokines such as IL-8 and IL-6 (Figures 6E and S4B). In addition to these findings, our result suggested that cleaved EFNA1 can still bind to EPHA2 effectively and may activate the kinase-dependent signaling, although not as efficiently as EFNA1-Fc or cell surface-bound EFNA1.

It has been reported that EFNA1 undergoes shedding by matrix metalloproteases (MMP-1, 2, 9, 13), disintegrin, and metalloproteinase (ADAM12, 17) in cancer cells.^25^^,^^26^^,^^27^^,^^28^ Additionally, ADAM10 contributes to the shedding of EFNA5 and EFNB2.^29^^,^^30^ Thus, we next investigated whether MMP/ADAM family proteases are involved in the FLA-PA-induced EFNA1 release. As expected, the broad MMP/ADAM inhibitors Batimastat^31^ reduced the FLA-PA-induced EFNA1 release in a concentration-dependent manner (Figures 6F and 6G). 100 μM O-phenanthroline (OPN)^32^ slightly suppressed FLA-PA induced EFNA1 release; however, higher concentrations of OPN induced EFNA1 secretion in the absence of FLA-PA, and further EFNA1 secretion was not induced by FLA-PA treatment (Figures 6F and 6G). On the other hand, TAPI-1 or TAPI-2,^33^^,^^34^ inhibitors of MMP-2,9 and ADAM17, which are involved in the shedding of EFNA1 and EFNA5, did not inhibit the FLA-PA-induced EFNA1 release (Figure 6F). In addition, Batimastat suppressed the EFNA1 release in the absence of FLA-PA, indicating that MMP/ADAM proteases could regularly release EFNA1 extracellularly in the absence of PAMPs (Figures 6F and 6G). Shedding of EFNA1 by MMPs requires the 175-181aa (V-L-H-S-I-G/A-H) region just before the GPI-anchor and MMPs/ADAM12 cleaves EFNA1 at R174 residue.^26^^,^^28^ To investigate whether the reduction in the EFNA1 p.m. expression by FLA-PA is due to shedding by the MMP/ADAM family, Sm-Flag-EFNA1-TEV (SF-a1-TEV) was constructed by replacing the 175–181 aa sequence of EFNA1 with the Tobacco Etch Virus (TEV) protease recognition sequence (E-N-L-Y-F-Q-G) and stably expressed in BEAS-2B cells (Figure 6H). The substitution of the TEV recognition sequence into Sm-Flag-EFNA1 did not affect the membrane localization of EFNA1 (Figure S6A). While FLA-PA increased the SF-a1 release and decreased the SF-a1 p.m. level, these effects were abolished in SF-a1-TEV (Figures 6I and 6J). In addition, the lower EFNA1 release and higher EFNA1 p.m. level were detected in SF-a1-TEV in the absence of FLA-PA (Figures 6I and 6J). These results indicate that the cleavage by MMP/ADAM proteases determines the EFNA1 releasing which is further promoted by the FLA-PA-TLR5 signaling pathway.

The single cell RNA sequence result in human bronchial epithelial cell showed that EFNA1-expressing cell populations expressed MMP14, 15, and ADAM9, 15 (Figure S6B).^35^ We thus evaluated the FLA-PA-induced Sm-Flag-EFNA1 release after KD of these candidate MMP/ADAM proteases including ADAM12. KD of either ADAM9, 12 or MMP15 suppressed the FLA-PA-induced Sm-Flag-EFNA1 release, indicating that ADAM9,12 and MMP15 could be responsible for the EFNA1 shedding in response to FLA-PA stimulation (Figures 6K–6S6D). We confirmed that ADAM9 KD completely, while ADAM12 KD slightly, reduced endogenous EFNA1 release induced by FLA-PA treatment (Figure 6L). These results suggest that while ADAM12 and MMP15 are also involved in EFNA1 shedding, the function of ADAM9 is crucial under physiological conditions. ADAM9 KD not only inhibited the shedding of EFNA1 but also suppressed the reduction in FLA-PA-induced EPHA2-EFNA1 *trans*-binding (Figure 6M). These results indicate that during FLA-PA stimulation, the decrease in EPHA2-EFNA1 *trans-*binding through TLR5-NF-κB could be underpinned by the shedding of EFNA1 mediated at least in part by ADAM9.

### The reduction of EPHA2-EFNA1 *trans*-binding rather than EFNA1 release is sufficient for inducing the inflammatory response independent of pathogen-associated molecular patterns

**Figure 7:**
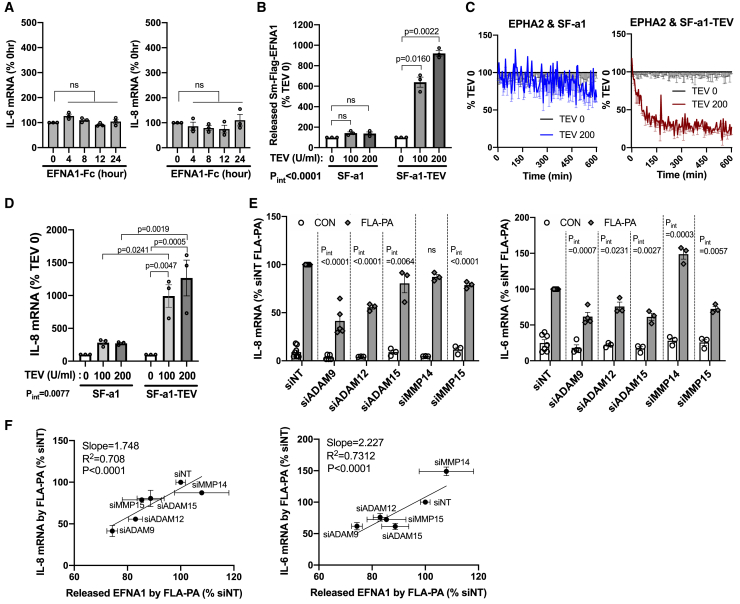
Cleavage of EFNA1 induces inflammatory response in AECs (A) The effect of soluble EFNA1 on cytokine mRNA expression in AECs. BEAS-2B cells were treated with 50 ng/mL EFNA1-Fc for 0–24 h, and the inflammatory response was analyzed by RT-qPCR. *n* = 3, RM one-way ANOVA, Dunnett’s test. (B) TEV protease-induced specific cleavage of SF-a1-TEV in cells. BEAS-2B cells expressing SF-a1 or SF-a1-TEV were treated with the indicated concentrations of TEV protease in CO2-independent medium and TEV buffer at 37°C for 3 h. The released SF-a1 and SF-a1-TEV in the supernatant were measured. *n* = 3, RM two-way ANOVA, Sidak’s test. (C) TEV protease-induced specific dissociation of EPHA2 and SF-a1-TEV in cells. iLg-EPHA2-HA expressing cells were co-cultured with SF-a1 or SF-a1-TEV expressing cells, and the EPHA2-EFNA1 *trans*-binding level was measured during treatment of 200 U/ml TEV protease. *n* = 3. (D) The impact of specific cleavage of SF-a1-TEV on inflammatory response in AECs. SF-a1 or SF-a1-TEV expressing cells were treated with the indicated concentration of TEV protease for 3 h, and the inflammatory response was analyzed by RT-qPCR. *n* = 3, two-way ANOVA, Tukey’s test. (E) The effect of MMPs/ADAMs KD on FLA-PA-induced inflammatory cytokine up-regulation. BEAS-2B cells expressing Sm-Flag-EFNA1 were transfected with 20 nM siRNA targeting ADAMs and MMPs, and the inflammatory response was analyzed by RT-qPCR 4 h after treatment with 100 ng/mL FLA-PA. siNT: *n* = 8, siADAM9: *n* = 5, Other groups: *n* = 3, RM two-way ANOVA, Sidak’s test. (F) Correlation analysis between the impact of ADAM/MMP KD on FLA-PA-dependent IL-8 and IL-6 mRNA induction (Figure 7E) and EFNA1 release (Figure S6C). Data are presented as mean ± SEM (A, B, C (-SEM only), D, E, F).

We have shown that FLA-PA induces the ADAM9-mediated EFNA1 shedding and reduced the EPHA2-EFNA1 *trans*-binding, leading to amplified inflammatory response in AECs. Since the EFNA1 release coincided with the reduced EPHA2-EFNA1 *trans*-binding upon FLA-PA treatment, it remains unclear whether the released soluble-EFNA1 (sEFNA1) or the reduced *trans*-binding enhances the inflammatory response. Previous studies using primary human sinonasal epithelial cells showed that 24-h stimulation with sEFNA1 induces the secretion of various inflammatory cytokines.^36^ Therefore, we examined whether the addition of recombinant human EFNA1-Fc (C-terminus IgG_1_ Fc Tag fused) as a sEFNA1 is sufficient to induce the inflammatory response in BEAS-2B cells. Even at concentrations that prompted inflammatory responses in sinonasal epithelial cells,^36^ EFNA1-Fc exhibited no impact on the induction of IL-8 and IL-6 mRNA in BEAS-2B cells (Figure 7A). This result suggests that the released sEFNA1 could not trigger an inflammatory response in AECs. Next, we examined whether EFNA1 cleavage itself induces inflammatory responses. We artificially induced EFNA1 shedding by treating SF-a1-TEV-expressing BEAS-2B cells with purified TEV protease (Figure 7B). TEV protease induced the EFNA1 release in SF-a1-TEV cells in a dose-dependent manner, whereas SF-a1 cells did not exhibit this response (Figure 7B). Furthermore, TEV protease notably diminished the *trans-*binding of EPHA2 with SF-a1-TEV cells, but not with SF-a1 cells, confirming the specific shedding and reduction of *trans-*binding (Figure 7C). RT-qPCR analysis showed that TEV protease treatment robustly increased IL-8 mRNA levels in SF-a1-TEV cells compared to SF-a1 cells, indicating that the shedding-induced reduction of the EPHs-EFNA1 binding is sufficient to induce inflammatory responses even in the absence of PAMPs (Figure 7D).

Finally, we investigated whether the ADAM9-induced EFNA1 shedding enhances the inflammatory response triggered by FLA-PA. In line with the inhibitory effect on the EFNA1 release, ADAM9 KD remarkably reduced the FLA-PA-induced IL-8 and IL-6 induction (Figure 7E). ADAM12 KD had a modest effect, while the KD of ADAM15 or MMP15 partially suppressed the FLA-PA-induced IL-8 induction, likely involving mechanisms distinct from EFNA1 shedding (Figure 7E). Remarkably, the FLA-PA-induced release of EFNA1 exhibited a strong correlation with the induction of IL-8 and IL-6 by FLA-PA, with both primarily regulated by ADAM9 (Figure 7F). These findings support the our hypothesis that the shedding of EFNA1 diminishes the *trans-*binding between EPHA2 and EFNA1, thereby inducing inflammatory response in AECs.

## Discussion

In this study, we developed a methodology to quantitatively monitor the *trans*-binding changes between EPH receptors and EFN ligands within live cells. We discovered that the *trans*-binding of EPHA2 and EFNA1 is temporary reduced upon exposure to various PAMPs associated with airway infection. Mechanistically, FLA-PA induced the EFNA1 shedding primarily mediated by ADAM9 through the TLR5-NF-κB pathway. This leads to a reduction in the *trans-*binding between EPHA2 and EFNA1, activating the EPHA2 ligand-independent pathways that likely amplify the inflammatory response for the effective elimination of the pathogen (Figure S8).

### The role of EPHA2 signaling in airway epithelial inflammatory responses

In this study, we show that the reduction in EPHA2-EFNA1 *trans-*binding is correlated with the intensity of the cellular inflammatory response. We discovered that EPHA2 promotes the acute-phase inflammatory response in the airway by assessing inflammation induction at 6 h after *P. aeruginosa* infection or FLA-PA stimulation. In line with these results, the employment of EPHA2 KO mice in a Bleomycin (BLM)-induced lung injury model has shown that EPHA2 not only enhances vascular permeability but also fosters the inflammatory response.^37^ Given that the activation of the NF-κB pathway is involved in BLM-induced lung injury,^38^ there is a potential implication that the mechanism of inflammation amplification mediated by EPHA2 through EFNA1 shedding could be relevant to the pathogenesis of inflammatory lung diseases. Conversely, the suppression of the early-stage inflammatory response observed in EPHA2 KO models may potentially impede the recruitment of immune cells during pathogen infections (Figures 1B and 1C),^37^ which could subsequently hinder pathogen clearance and promote long-term tissue damage. Indeed, in the context of lung inflammation caused by *Mycoplasma pulmonis* infection, tissue damage beyond 14 days post-infection is exacerbated in EPHA2 KO mice compared to WT mice.^39^ Hence, it is imperative to differentiate between the roles of EPHA2 in the early-phase airway inflammatory response and the subsequent inflammatory pathogenesis driven by immune cells. In lung tissue, multiple cell subsets consist heterogenic environment with differing expression patterns of TLRs, EPH, and EFN coexist alongside immune cells. The correlation observed in Beas2B cells between inflammation induction by PAMPs treatment, EPHA2-EFNA1 dissociation, and EFNA1 shedding suggests that signals through TLR2, 3, 4, and 5 may also drive EPHA2-EFNA1 dissociation in accordance with the strength of the inflammatory response (Figures 4D and 6E). Therefore, unlike the findings from our *in vitro* studies, the effects of infectious stimuli in lung tissue are likely regulated at the level of specific cell subsets and intercellular interactions.

EPHA2 has also been reported to exhibit anti-inflammatory effects on inflammation associated with ovalbumin-induced acute asthma models or reovirus infection.^40^ In this scenario, it was demonstrated that tyrosine residue-phosphorylated EPHA2 inhibits the inflammasome through NLRP3 phosphorylation, indicating that the ligand-dependent activation of EPHA2 may attenuate airway inflammation.^40^ This suggests that the EPHA2 ligand-dependent pathway could be constitutively activated in AECs forming the monolayer to suppress the inflammatory response.

In this study, we have demonstrated that FLA-TLR5 signaling reduces EPHA2-EFNA1 *trans*-binding and increases the phosphorylation of EPHA2 at S897 (Figures 5B–5D). Since we have no direct evidence showing that the reduces EPHA2-EFNA1 *trans*-binding by FLA treatment led to the increased S897 phosphorylation of EPHA2, it is possible that parallel pathways activated downstream of TLR5 could independently lead to EPHA2 S897 phosphorylation and EFNA1 cleavage. Recently, it has been reported that the ligand-free EPHA2 assembles into multimers and becomes a substrate for multiple serine-threonine kinases that phosphorylate EphA2 on S897.^41^ Thus, it is likely that ligand-free EPHA2, resulting from EFNA1 cleavage, promotes EPHA2 S897 phosphorylation. The phosphorylation of EPHA2 at S897 has been reported to be promoted by Akt and ERK-RSK signaling.^11^^,^^42^ Since these MAPK signaling pathways are activated during various PAMPs stimulations, it is plausible that the EPHA2 dissociation from the ligand due to EFNA1 cleavage, in coordination with the activation of these MAPK pathways, leads to the activation of the EPHA2 ligand-independent pathway. On the other hand, EPHA2 S897 phosphorylation has been reported to induce the activation of Akt, STAT3 signaling, and the activation of Rac1 through the Ephexin4-RhoG pathway.^24^^,^^43^ Considering that Akt and Rac1 have been reported to function in an activating manner toward the NF-κB pathway,^44^^,^^45^ activation of the EPHA2 ligand-independent pathway during airway inflammation may amplify the inflammatory response through these pathways to the NF-κB pathway, which regulates the transcriptional expression of inflammatory cytokines.

The occurrence of EFNA1 shedding during inflammation suggests that EFNA1 reverse signaling may be suppressed. Although EFNA5 reverse signaling involving tyrosine kinases such as FYN^46^ has been reported, its role in inflammatory pathways remains unclear. EFNA1 has a potential to *trans*-bind with EPHA4 that abundantly expressed in BEAS-2B cells (Figures 2A and 3G). EFNA1 reverse signaling should be maintained with EPHA4 even under EPHA2 KD conditions. Therefore, if EFNA1 reverse signaling were the dominant pathway for the inflammatory response, EPHA2 and EFNA1 KD would be expected to exhibit a greater anti-inflammatory effect compared to EPHA2 single KD. On the other hand, since the combination of EFNA1 KD with EPHA2 KD did not result in further anti-inflammatory effects (Figure 2G), we propose that the pathway mediated by EPHA2 primarily contributes to the inflammation induced by FLA-PA.

### Mechanism of the EPHA2-EFNA1 *trans*-binding perturbation

It has been reported that soluble EFNB2 released through shedding mediated by ADAM10 is involved in pulmonary fibrosis, and EFNA1 released by ADAM12, which is activated by TGF-β stimulation, promotes cancer metastasis to the lungs through EPHA1.^27^^,^^30^ In these reports, soluble EFNs act on EPHs that are expressed in neighboring cells in an autocrine or paracrine manner, leading to cellular responses. However, in this study, the treatment of EFNA1-Fc did not induce an inflammatory response in BEAS-2B cells (Figure 7A). Based on our results, the dissociation of EPHA2-EFNA1 *trans-*binding, rather than the released EFNA1, may be crucial for the amplification of inflammation in AECs (Figure 7D). Recently, it has been shown that ionizing the irradiation-induced activation of ADAM17 in lung cancer cells triggers EFNA1 shedding. This, in turn, promotes EPHA2 phosphorylation at S897 and cancer cell migration. Similar to our study, it has been found that stimulus-dependent EFNA1 shedding activates the EPHA2 ligand-independent signaling pathway.^25^ We observed that FLA-PA-induced EFNA1 shedding still occurred even when using an ADAM17 inhibitor (Figure 6F). This suggests that, contrary to previous assumptions, the disruption of EPHA2-EFNA1 *trans-*binding during inflammation primarily depends on ADAM9 (Figures 6K and 6L). In addition to the functional activation of ADAM9, inflammatory stimulation-induced changes in cell morphology might also influence the *trans-*binding between EPHA2 and EFNA1. In our experimental conditions, FLA-PA treatment did not lead to significant alterations in BEAS-2B morphology, but in some instances, shrunken cells exhibited restricted EPHA2-EFNA1 co-localization (Figures S3D and S3E). Our finding that ADAM9 KD prevented the FLA-PA-induced reduction in EPHA2-EFNA1 *trans*-binding suggests that ADAM9 primarily contributes to the dissociation of EPHA2-EFNA1 (Figure 6M). However, further investigation is required to elucidate additional aspects of the dissociation mechanism. While the role of ADAM9 in EFNA1 shedding has not been specifically reported, there is evidence demonstrating that ADAM9 plays a role in the shedding of EFNB1 and EFNB2, which in turn activates the Akt pathway in colorectal cancer.^47^ Bacterial LPS activates the ADAM9-dependent shedding of angiotensin-I converting enzyme ACE in endothelial cells.^48^ ADAM9 activity is also enhanced in the presence of reactive oxidative species which could be produced during lung inflammation.^49^^,^^50^

The complete prevention of EFNA1 shedding upon FLA-PA treatment by NF-κB and TAK1 inhibitors suggests that the activation of ADAM9 could be mediated through an NF-κB-dependent pathway. While the involvement of the NF-κB pathway in ADAM9 activation has not been demonstrated, our observation is consistent with the fact that ADAM9 can be activated by LPS, a well-known inducer of NF-κB activation.^48^^,^^51^ Additionally, NF-κB signaling has been reported to promote the maturation of ADAM17,^52^ and it may also play a role in the maturation of ADAM9. The activation of ADAM9 through the NF-κB pathway could potentially explain why various PAMPs, which activate NF-κB via toll-like receptors, lead to the EFNA1 shedding and disruption of the EPHA2-EFNA1 *trans*-binding (Figures 6D and 4A).

### Targeting the EPH-EFN *trans*-binding and ADAM9 in airway epithelial cells inflammation

The present study shows that the suppression of EPHA2, A4, EFNA1, and ADAM9 expression reduced the PAMPs-induced inflammatory response of AECs. This suggests that these molecules could serve as targets for anti-inflammatory therapy in diseases such as chronic obstructive pulmonary disease (COPD), where excessive inflammation in the airway epithelium accelerates pathology. In particular, the reduced EPHA2-EFNA1 *trans*-binding during pathogen infections promotes inflammatory responsiveness, and the EPHA2 ligand-independent pathway may underlie this mechanism. Indeed, the activation of the EPHA2 ligand-independent pathway is a primary target in malignant cancer therapy, and peptides and enzyme inhibitors targeting EPHA2 have already been developed.^53^ Further analysis has the potential to expand the application of these compounds to respiratory inflammations diseases.

It has been reported that ADAM9 is associated with airway inflammation and infection. ADAM9 level is increased in patients with COPD, and strategies to inhibit ADAM9 activity or reduce its expression in the lung are proposed to limit the progression of several key aspects of COPD.^54^ In COPD pathology, the amount of ADAM9 in sputum is correlated with IL-8 production, and the augmented expression of ADAM9 on the surface of airway cells is associated with emphysema and airway remodeling.^55^ Increased expression of ADAM9 in lung epithelial cells and macrophages has also been reported in a COPD mouse model, and the severity of COPD induced by tobacco smoke was milder in ADAM9-deficient mice than in WT mice.^56^ Additionally, the expression of ADAM9 on the membrane surface of neutrophils increases with degranulating agents, and an increase in neutrophil-derived ADAM9 expression has been reported in acute lung injury induced by LPS or bleomycin.^57^ Based on the results of this study, increased expression of ADAM9 in patients with COPD may contribute to the amplification of inflammatory responses by promoting the shedding of substrates such as angiogenic factors^56^ and soluble IL-11 receptor, as well as EFNA1. This shedding exacerbates the EPHA2-EFNA1 dissociation, possibly contributing to the acquisition of excessive inflammatory responses and neovascularization. Therefore, the specific inhibition of ADAM9 under inflammatory stimulation may protect the EPHA2-EFNA1 *trans*-binding and attenuate inflammation. The development of ADAM9 inhibitors has a potential to create the anti-inflammatory therapy in pulmonary disease.^58^ The inflammation amplification mechanism through the EPHA2-EFNA1 *trans*-binding could be a drug target to regulate the respiratory inflammation without causing excessive immune suppression.

### Limitations of the study

This study demonstrated that EFNA1 cleavage mediated by ADAM9 and the subsequent dissociation of EPHA2-EFNA1 binding occur in airway epithelial cells in response to various PAMP stimuli. Additionally, it was shown that inflammatory cytokine induction by FLA-PA is suppressed in both mouse models and cell lines under conditions of EPHA2 KO or KD of EPHA2, EFNA1, and ADAM9. Artificial EFNA1 cleavage was also shown to induce inflammatory responses in BEAS-2B cells independent of PAMP stimuli. However, it has not been directly demonstrated whether the dissociation of EPHA2-EFNA1 interactions contributes to the promotion of inflammatory responses in actual inflammatory conditions. Furthermore, FLA-PA stimulation was confirmed to increase EPHA2 S897 phosphorylation in a TLR5-dependent manner. Given that EFNA1 cleavage depends on ADAM9 activated via TLR5 signaling, it remains unclear whether the transient increase in S897 phosphorylation is caused by the dissociation of EFNA1 binding or mediated via the MAPK pathway downstream of TLR5 signaling. Regarding the NanoBiT system used to measure EPH-EFN *trans*-binding, it should be noted that LgBiT and SmBiT tags are fused upstream of the ligand- or receptor-binding domains of EPH or EFN. This may limit the ability to fully replicate physiological binding, necessitating the careful interpretation of the results. Moreover, the effects of sequestration or cell repulsion between EPH-expressing and EFN-expressing cells, which may arise during co-culture, should be considered. While this system is suitable for analyzing relative changes in binding levels of each EPH-EFN pair in response to stimulation, it is not appropriate for comparing binding affinities between different EPH-EFN pairs.

## Resource availability

### Lead contact

Further information and requests for resources and reagents should be directed to and will be fulfilled by the the lead contact, Ryosuke Fukuda (ocf2ocf2@gmail.com).

### Material availability

All unique/stable reagents generated in this study are available from the lead contact with a completed materials transfer agreement.

### Data and code availability

• Data: All data and any additional information required to reanalyze the data reported in this article will be shared by the lead contact upon request. Original western blot images have been deposited at Mendeley and are publicly available as of the date of publication. Accession number is listed in the key resources table. Microscopy data reported in this article will be shared by the lead contact upon request.
• Code: This article does not report original code.

## Acknowledgments

We thank Laboratory Animal resource Center of Tsukuba University for the establishment of Epha2 KO mouse on the program (PL21-03). We also thank Okiyoneda lab members (Kwansei Gakuin University, Japan) for technical supports and M. A. Suico (Kumamoto University, Japan) for proof reading of the article.

The authors gratefully acknowledge the financial support from the 10.13039/501100001691JSPS KAKENHI Grant Number 21K15475, 19K16508 and JP 16H06277 (CoBiA) to R.F., and individual special research subsidy with grants from 10.13039/100012044Kwansei Gakuin University to R.F. and T.O.

## Author contributions

Conceptualization, R.F.; methodology, R.F.; validation, R.F.; formal analysis, R.F.; investigation, R.F., S.B., and D.H.; writing – original draft, R.F.; writing – review and editing, R.F., Y.K., and T.O.; visualization, R.F., and Y.K.; supervision, R.F., and T.O.; and funding acquisition, R.F. and T.O.

## Declaration of interests

The authors declare no competing interests.

## Declaration of generative AI and AI-assisted technologies in the writing process

During the preparation of this work the authors used ChatGPT in order to improve language and readability. After using this tool/service, the authors reviewed and edited the content as needed and take full responsibility for the content of the publication.

## STAR★Methods

### Key resources table

**Table:** 

REAGENT or RESOURCE	SOURCE	IDENTIFIER
**Antibodies**		

Anti-HA.11 Epitope Tag Antibody	Biolegend	Cat# MMS-101R; RRID: AB_291262
Anti-FLAG M2 antibody	Cell Signaling Technology	Cat# 14793; RRID: AB_2572291
Phospho-EphA2 (Ser897) (D9A1) Rabbit mAb	Cell Signaling Technology	Cat# 6347; RRID:AB_11220420
Phospho-EphA2 (Tyr772) Antibody	Cell Signaling Technology	Cat# 8244; RRID:AB_10860415
Anti-DYKDDDDK tag Monoclonal Antibody, Unconjugated, Clone 1E6	FUJIFILM Wako Pure Chemical Corporation	Cat# 014–22383; RRID:AB_10659717
EphA2 Antibody (C-3)	Santa Cruz Biotechnology	Cat# sc-398832; RRID: AB_3665708
Anti-ephrin-A1 Antibody (A-5)	Santa Cruz Biotechnology	Cat# sc-377362; RRID:AB_2893328
Na+/K + -ATPase alpha3 (H-4)	Santa Cruz Biotechnology	Cat# sc-365744; RRID:AB_10848453
Alexa Fluor 488-AffiniPure Donkey Anti-Mouse IgG (H + L)	Jackson ImmunoResearch Labs	Cat# 715-545-150; RRID:AB_2340846
Alexa Fluor 594-AffiniPure Goat Anti-Rabbit IgG (H + L)	Jackson ImmunoResearch Labs	Cat# 111-585-003; RRID:AB_2338059

**Bacterial and virus strains**		

Pseudomonas aeruginosa (Schroeter 1872) Migula 1900	RIKEN BRC (Japan)	JCM #14847

**Chemicals, peptides, and recombinant proteins**		

Lipopolysaccharides from Pseudomonas aeruginosa 10	Sigma-Aldrich	Cat# L9143
Lipopolysaccharides from Salmonella enterica serotype enteritidis	Sigma-Aldrich	Cat# L7770
Lipopolysaccharides from Escherichia coli O111:B4	Sigma-Aldrich	Cat# L2630
Lipoteichoic acid from Staphylococcus aureus	Sigma-Aldrich	Cat# L2515
PGN-SA	InvivoGen	Cat# tlrl-pgns2
Pam3CSK4	InvivoGen	Cat# tlrl-pms
FLA-PA Ultrapure	InvivoGen	Cat# tlrl-pafla
Poly(I:C) (HMW)	InvivoGen	Cat# tlrl-pic
Heat Killed Staphylococcus aureus	InvivoGen	Cat# tlrl-hksa
Heat Killed Pseudomonas aeruginosa	InvivoGen	Cat# tlrl-hkpa
Heat-killed C. albicans	InvivoGen	Cat# tlrl-hkca
MG-132	Funakoshi	Cat# CS-0471
Bafilomycin A1	Funakoshi	Cat# CAY-11038
Wedelolactone	MedChemExpress	Cat# HY-N0551; CAS: 524-12-9
Takinib	MedChemExpress	Cat# HY-103490; CAS: 1111556-37-6
*o*-Phenanthroline	APExBIO	Cat# B7854; CAS: 66-71-7
Batimastat	Cayman Chemical	Cat# 14742; CAS: 130370-60-4
TAPI-1	APExBIO	Cat# B4686; CAS: 171235-71-5
TAPI-2	Cayman Chemical	Cat# 14695; CAS: 187034-31-7
TEV Protease	NEW ENGLAND Biolabs	Cat# P8112S
Recombinant Human Ephrin-A1 Fc Chimera Protein, CF	R&D systems	Cat# 6417-A1

**Critical commercial assays**		

The Mouse CXCL1/KC DuoSet ELISA	R&D systems	Cat# DY453-05
ReverTra Ace qPCR RT Master Mix	TOYOBO	Cat# FSQ-301
Thunderbird SYBR qPCR Mix	TOYOBO	Cat# QPS-201
Nano-Glo® Live Cell Assay System	Promega	Cat# N2011
Nano-Glo® Endurazine™ Live Cell Substrates	Promega	Cat# N2571

**Deposited data**		

Raw WB data images	This paper	Mendeley Data https://doi.org/10.17632/hcgjnsw44k.1

**Experimental models: Cell lines**		

BEAS-2B	ECACC	Cat# 95102433

**Experimental models: Organisms/strains**		

Mouse: Epha2 KO: B6, Epha2^−/−^/J	This paper	None

**Oligonucleotides**		

Primers for Q-RT-PCR, see Table 2	This paper	N/A
Silencer® Select siRNA Human EPHA2	Thermo Fisher Scientific	Cat# s4564
Silencer® Select siRNA Human EPHA2	Thermo Fisher Scientific	Cat# s4565
Silencer® Select siRNA Human EPHA2	Thermo Fisher Scientific	Cat# s4566
Silencer® Select siRNA Human EFNA1	Thermo Fisher Scientific	Cat# s4498
Silencer® Select siRNA Human ADAM9	Thermo Fisher Scientific	Cat# s16684
Silencer® Select siRNA Human ADAM12	Thermo Fisher Scientific	Cat# s15575
Silencer® Select siRNA Human ADAM15	Thermo Fisher Scientific	Cat# s16682
Silencer® Select siRNA Human MMP14	Thermo Fisher Scientific	Cat# s8877
Silencer® Select siRNA Human MMP15	Thermo Fisher Scientific	Cat# s8881
EPHA2 gRNA: TCACACACCCGTATGG CAAAGGG	This paper	N/A
TLR5 gRNA: TTGATGGCCGAATAGCCTTT	This paper	N/A
EPHA2 DNA sequencing for KO checkFw: AGAGTTGGGGTTCCTGGAGGGATCRv: GCTGAGCTGCTGAATTGAAGCCAG	This paper	N/A
TLR5 DNA sequencing for KO checkFw: GTCTGTATCTGCCAACAGCCACGTCRv: AGCATCTGGATGCAAGAAGTATATC	This paper	N/A

**Recombinant DNA**		

pLIX402	Addgene	#41394
pLX304	Addgene	#25890
EPH and EFN original plasmid list, see Table 1	DNASU/Addgene	Table 1
pLIX402-LgBiT-EPHA2-HA	This paper	N/A
pLIX402-LgBiT-EPHA4-HA	This paper	N/A
pLIX402-LgBiT-EPHB1-HA	This paper	N/A
pLIX402-LgBiT-EPHB3-HA	This paper	N/A
pLIX402-LgBiT-EPHB4-HA	This paper	N/A
pLIX402-LgBiT-EPHB6-HA	This paper	N/A
pLX304-SmBiT-Flag-EFNA1	This paper	N/A
pLX304-SmBiT-Flag-EFNA5	This paper	N/A
pLX304-SmBiT-Flag-EFNB1-V5	This paper	N/A
pLX304-SmBiT-Flag-EFNB2-V5	This paper	N/A
pLX304-SmBiT-Flag-EFNA5 RBD-EFNA1	This paper	N/A
pLX304-SmBiT-Flag-EFNA1 RBD-EFNA5	This paper	N/A
pLX304-SmBiT-Flag-EFNA1-TEV	This paper	N/A

**Software and algorithms**		

ImageJ Fiji	ImageJ	https://imagej.net/ij/
Prism 8	GraphPad	https://www.graphpad.com/

### Experimental model and study participant details

#### Mammalian cell line

The human bronchial epithelial cell line BEAS-2B cells were obtained from The European Collection of Authenticated Cell Cultures (ECACC) and cultured in DMEM high-glucose medium supplemented with 10% FBS and Penicillin/Streptomycin.^59^

#### Mouse

C57BL/6J (WT) mice were obtained from Japan SLC, Inc. C57BL/6J background Epha2 KO mice were established by Laboratory Animal Resource Center (Tsukuba University). For generation of Epha KO mice by CRISPR/Cas9-mediated gene editing, we excluded exon 6 of *Epha2 by* targeting introns both side of Exon 6: 5′- TAAAGACGGAAGTCGACATCAGG -3′ and 5′-GAGGGGGACTATACATCCCCAGG -3’. Genome sequence of Epha2 KO mice was shown in Figures S4A and S4B. EPHA2 +/− mice were established by crossing WT and EPHA2 KO mice. Both male and female littermate EPHA2 WT, +/−, and KO mice, which were obtained by mating EPHA2 +/− mice, were used for experiments. Genotyping of mouse strain was performed by PCR amplification. Male and female mice were bred in a specific pathogen-free environment and used for experiments at 10–11 weeks of age. The impact of sex was not examined, which may pose a limitation to the generalizability of the research findings.

For intratracheal administration, a mixture of three anesthetics, midazolam, butorphanol tartrate, and medetomidine hydrochloride (Wako, Japan), was administered intraperitoneally, followed by intratracheal administration of 50 μL of the solution through a tracheal catheter. For intranasal infection, 2 μL of *P. aeruginosa* in PBS was alternately added to the left and right nasal cavities at 1-min intervals under anesthetized condition, until achieving 50 μL of the solution (1 × 10^9^ CFU) per mouse. After administration, the mice were awakened with atipamezole hydrochloride (Wako, Japan) and sacrificed 6 h later. BALF was collected using 1.5 mL of PBS and centrifuged at 12,000 rpm for 5 min. Supernatant was collected and used for KC ELISA. The lungs were collected after perfusing for WB, HE-staining and RNA extraction for RT-qPCR using TRIzol (Thermo Fisher Scientific). All experimental procedures approved by the institutional animal use committees of Kwansei Gakuin University (protocol 2022-25).

### Method details

#### Constructs

The entry clones of EPHs and EFNs were purchased from DNASU and Addgene (Table 1). The LgBiT (182 aa)-linker (24 aa) and SmBiT (VTGYRLFEEIL, 11 aa)-Flag (8 aa)-linker (29 aa) sequences were inserted by In-Fusion method (Takara, Japan) just before the LBD of EPH receptors and RBD of EFN ligands, respectively. The lentiviral expression plasmids of Dox-inducible LgBiT-EPH-HA and constitutive SmBiT-Flag-EFNA, SmBiT-Flag-EFNB-V5 were established by LR reaction by using pLIX402 (Addgene #41394) and pLX304 (Addgene #25890) destination vectors and LR clonase II (Thermo Fisher, USA). The RBD regions of pLX-SmBiT-Flag-EFNA1 and EFNA5 were swapped, and the MMP recognition sequence was replaced with the TEV recognition sequence by the In-Fusion method.

#### Cell line experiments

BEAS-2B iLgBiT-EPH-HA, SmBiT-Flag-EFN (-V5), SmBiT-Flag-a5 RBD-EFNA1, SmBiT-Flag-a1 RBD-EFNA5 and SmBiT-Flag-EFNA1-TEV cells were generated by lentivirus transduction as previously described.^60^ Briefly, lentivirus was generated by transfecting HEK293T cells with lentiviral vectors carrying EPH or EFN gene sequences, pMD2.G (Addgene #12259), and psPAX2 plasmid (Addgene #12260) using PEI MAX (Polysciences, #24765). The collected medium containing lentivirus was used to infect BEAS-2B cells in the presence of 7 μg/mL polybrene (Sigma-Aldrich, #H9268). Stable cell lines were established through selection with 1 μg/mL puromycin (Sigma-Aldrich, #P9620). iLg-EPHA2-HA-expressing cells and Sm-Flag-EFN-expressing cells were enriched for populations with consistent expression levels by FACS (SONY, #SH800-S) using EPHA2 or Flag antibodies, respectively. These enriched cell populations were subsequently utilized in experiments. siRNA transfection in BEAS-2B cells was accomplished using Lipofectamine RNAiMax transfection reagent (Invitrogen). siRNA transfected cells were used for the experiments 3 days post-transfection. The treatment of BEAS-2B cells with pathogen factors, inhibitors, EFNA1-Fc, and *P. aeruginosa* was performed after incubation at 37°C for 2 h in CO_2_ independent medium without FBS, at the concentrations and dosing times indicated. Inhibitors were pretreated for 2 h and co-treated at the time of pathogen factor treatment.

#### Establishment of KO cell by CRISPR-Cas9 system

BEAS-2B EPHA2 KO and TLR5 KO cells were established as previously^61^ using the following gRNA.

EPHA2 gRNA: TCACACACCCGTATGGCAAAGGG

TLR5 gRNA: TTGATGGCCGAATAGCCTTT.

Knockout of each gene was confirmed by WB (except TLR5 KO) and genome DNA sequencing. Each gene’s genomic locus was amplified by PCR using following primers.

EPHA2: Fw 5′-AGAGTTGGGGTTCCTGGAGGGATC-3′, Rv 5′-GCTGAGCTGCTGAATTGAAGCCAG-3’

TLR5 Fw 5′-GTCTGTATCTGCCAACAGCCACGTC-3′), Rv 5′-AGCATCTGGATGCAAGAAGTATATC-3’

The PCR product was cloned into pMD20-T using a Mighty TA-cloning Kit (Takara Bio) and determined by DNA sequencing.

#### Cytokine ELISA

The Mouse CXCL1/KC DuoSet ELISA (R&D systems) kit was used for cytokine ELISA. Assays were performed according to the manufacturer’s protocol. SuperSignal West Pico Plus (Thermo Fisher Scientific) was used as the luminescent substrate and measurements were performed with Varioskan Flash (Thermo Fisher Scientific).

#### Hematoxylin eosin (HE) staining

The collected lung tissue was fixed by rotating overnight at 4°C in neutral buffered formalin (Wako, Japan). The lung tissue was then dehydrated in a 30% sucrose solution for 1–3 days at 4°C, embedded in OCT compound (Sakura Finetek, Japan), and sliced into 10 μm sections using a microtome. The sections were air-dried overnight at room temperature. Hematoxylin-Eosin staining was performed using a Hematoxylin-Eosin stain kit (COSMO BIO, Japan) according to the manufacturer’s protocol, and images were captured using an Axioplan 2 and AxioCam MRc (ZEISS). Image analysis was performed using Fiji software to calculate the number of cell nuclei per tissue area (Nuclei number/1 μm^2^).

#### Real time-quantitative PCR (RT-qPCR)

RNA recovery and RT-qPCR analysis were performed as described previously.^62^ Briefly, Total RNA was extracted from cells or tissues using TRIzol (Thermo Fisher, Waltham, MA, USA) according to the manufacturer’s protocols. An amount of 500 ng of total RNA was then used for the reverse transcription reaction using ReverTra Ace qPCR RT Master Mix (Toyobo, Japan). RT-qPCR was performed in the LightCycler 480 System (Roche Diagnostics, Switzerland), and the gene expression was examined by SYBR Advantage qPCR Premi Thunderbird SYBR qPCR Mix (Toyobo, Japan). GAPDH (human sample) and Gapdh (mouse sample) were used as the housekeeping gene. The sequences of primers used for quantitative RT-PCR are listed in Table 2.

#### Plasma membrane (PM) level measurement

The membrane expression level of iLg-EPH-HA was measured as the NanoBiT luminescence signal by adding mixture of 2 p.m. HiBiT-Halo (Promega) and NanoGlo substrate in opti-MEM to the cells. NanoBiT signal was measured 5 min after adding the mixture. Dox-untreated cells were used as background controls. The membrane expression level of SmBiT-Flag-EFN was measured using a cell-surface ELISA by using anti-Flag antibody (clone #1E6, Fujifilm) and HRP conjugated anti-Mouse IgG. BEAS-2B parental cells were used as background controls. The Cell-surface ELISA was performed as previously described.^60^ Measurements were performed using Varioskan Flash (Thermo Fisher Scientific).

#### EPH-EFN *trans*-binding measurement

Cultured BEAS-2B cells expressing iLgBiT-EPH-HA or SmBiT-Flag-EFN were trypsinized and mixed in a 1:1 ratio, then seeded into 96-well plates with various EPH-EFN patterns. Expression of iLg-EPH-HA was induced by treating the cells with Dox starting from the first day after seeding. The Dox concentrations used for each iLg-EPH-HA were modified to achieve equilibrate PM expression as follow, iLg-EPHA2 and A4 (50 ng/mL), B1 (500 ng/mL), B3 (150 ng/ml), B4 and B6 (1000 ng/mL). The Dox-untreated group was used as the background control. Each study was conducted starting from 48 h after Dox induction. NanoGlo Live cell assay (Promega) was used to evaluate EPHs-EFNs binding amount according to the manufacturer’s instructions. Measurements were performed using Varioskan Flash (Thermo Fisher Scientific). The *trans*-binding signal was obtained by subtracting the background signal of the -Dox group from the signal of the +Dox group, and then shown as a ratio with the EPHA2-EFNA1 signal set at 100%. For the Real-Time EPHs-EFNs *trans*-binding assay during pathogen factor treatment, NanoGlo Endurazine (Promega) was used. After Dox induction for 48 h, EPHs-EFNs mixed co-cultured cells were incubated in CO_2_ independent medium with NanoGlo Endurazine for 2 h at 37°C. The pathogen factors were then added at the final concentrations, and the measurement was started. NanoBiT signal was measured every 10 min using LUMINOSKAN (Thermo Fisher Scientific), and the binding ratio was calculated relative to CON value at each time point. An equivalent amount of solvent for each pathogenic factor or drug was treated for the CON group.

#### ImmunoFluorescence

Immuno-Fluorescence was performed as described previously.^61^ Briefly, BEAS-2B cells expressing iLg-EPH-HA and Sm-Flag-EFN were cultured on cover glass and iLg-EPH-HA expression was induced by treatment with 1 μg/mL Dox for 48 h. The cells were then fixed with 4% Paraformaldehyde and permeabilized with 0.1% Triton in PBS. After blocking with 1% BSA in PBS, the EPHs were detected using an anti-HA antibody (clone #16B12, Biolegend, 1:200 dilution) and Alexa Fluor 488 AffiniPure Donkey Anti-Mouse IgG (H + L) (Jackson Immuno Research, 1:500 dilution) and EFNs were detected with anti-Flag antibody (clone #D6W5B, Cell Signaling Technology (CST), 1:200 dilution) and Alexa Fluor 594 AffiniPure Goat Anti-Rabbit IgG (H + L) (Jackson Immuno Research, 1:500 dilution). Alexa Fluor 594 conjugated Wheat Germ Agglutinin (WGA) (Thermo Fisher Scientific) was used for PM staining and DAPI (Wako) was used for nucleus staining. Washes with 0.1% tween in PBS were performed between each step. The samples were mounted with VECTASHIELD (Vector Laboratories) and observed using a Leica SP-8 microscope.

#### Western Blotting (WB)

Western Blotting was performed as previously described.^60^ Cell and tissue samples were prepared by using RIPA buffer added protease inhibitors (5 μg/mL pepstatin A and 5 μg/mL leupeptin B) and phosphatase inhibitor (1μM Sodium fluoride, 25μM Sodium pyrophosphate, 1μM β-glycerophosphate, 1μM Sodium orthovanadate, for EPHA2 S897 phosphorylation detection) and Laemmli sample buffer. Only samples for EPHA4 detection were incubated at 95°C for 10 min to improve antibody binding. Cell supernatant was collected after centrifuge (12,000 rpm, RT, 5min) and proteins were precipitated with equal amount of 20% TCA (Trichloroacetate) 30 min on ice and centrifugation (15,000g, 4°C, 10min). Protein pellet was washed with ice-cold acetone and lysed in RIPA + Laemmli sample buffer. Lysate was mixed with Laemmli sample buffer after centrifugation (12,000 rpm, 4°C, 15min). After SDS page, the protein was transferred to Nitrocellulose membrane. The amounts of transferred proteins confirmed by Ponceau staining and membrane was blocked with 5% skim milk in 0.1% tween-PBS. TBS was used instead of PBS for the detection of phosphorylated proteins. The antibodies used for detection are as follows. HA (clone #16B12, Biolegend, 1:1000 dilution), Flag (clone #1E6, Fujifilm, 1:1000 dilution), EPHA2 (clone #C-3, Santa Cruz Biotechnology (SCB), 1:2000 dilution), EFNA1 (clone #A-5, SCB, 1:200 dilution), Na^+^/K^+^ ATPase (clone #H-3, SCB, 1:10000 dilution), phospho-S897 EPHA2 (clone #D9A1, CST, 1:500 dilution), phospho-Y772 EPHA2 (#8244, CST, 1:500 dilution). Fusion (Vilber Bio Imaging) was used for detection and quantification.

#### *Pseudomonas aeruginosa* infection

*P. aeruginosa* (JCM #14847) was obtained from RIKEN BRC (Japan). CFU and MOI of *P. aeruginosa* were calculated from OD600 values.^63^ The required amount of *P. aeruginosa* was suspended in PBS after centrifugation and used for *in vitro* and *in vivo* experiments. Experiments using *P. aeruginosa* were conducted in a Biosafety Level 2 (BSL2) facility under the approval of the Regulations for the Safe Management of Recombinant DNA Experiments at Kwansei Gakuin University.

#### Released SmBiT-flag-EFNA1 measurement

The amount of SmBiT-Flag-EFNA1 released into the medium was measured by the NanoBiT method using iLg-EPHA2-HA-expressing BEAS-2B cells. SmBiT-Flag-EFNA1-expressing BEAS-2B cells were cultured in a CO_2_ independent medium containing various inhibitors for 2 h and then replaced with a new CO_2_ independent medium containing the inhibitors and FLA-PA or TEV protease and TEV buffer (New England BioLabs). After treatment, the medium was centrifuged (6,000 rpm, room temperature, 5 min) and the supernatant was collected. NanoGlo substrate and buffer were added to the collected supernatant and reacted with BEAS-2B cells that had been induced to express iLgBiT-EPHA2-HA by 1 μg/mL Dox for 2 days on 96-well plates. After 10 min of reaction, luminescence was measured by Varioskan Flash and the relative amount of SmBiT-Flag-EFNA1 released into the supernatant was calculated. The supernatant from BEAS-2B parental cells was used as a background control.

### Quantification and statistical analysis

The comparison between two groups was performed using an unpaired *t-*test (two-tailed) or an unpaired multiple *t-*test (Bonferroni-Dunn test). One-way ANOVA was used for multiple group comparisons for one factor. two-way ANOVA was used for multiple group comparisons for two factors. *p* values are shown in each figure. All experiments were repeated at least three times to confirm reproducibility, and the biological replicate (n) of each experiment was noted in figure legends. Error bars represent the values of the mean SEM. Statistical analysis and figure creation were performed with GraphPad Prism 8.
