# Supplementary material for: Perturbation of EPHA2 and EFNA1 trans binding amplifies inflammatory response in airway epithelial cells
Source: iScience. 2025 Jan 22;28(2):111872. doi: 10.1016/j.isci.2025.111872 (PMC11847143; doi:10.1016/j.isci.2025.111872)
Supplement: Document S1. Figures S1–S8 [file mmc1.pdf]

iScience, Volume *28*

Supplemental information

Perturbation of EPHA2 and EFNA1 *trans* binding amplifies inflammatory response in airway epithelial cells

Ryosuke Fukuda, Shiori Beppu, Daichi Hinata, Yuka Kamada, and Tsukasa Okiyoneda

Figure S1

A

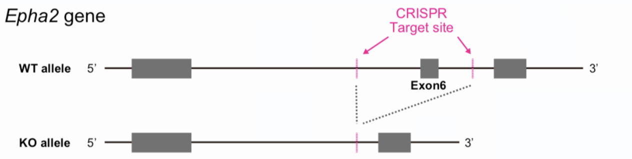

B

Epha2 (Genome)

```
WT  CTGTCTCAAGAAGATAAAGTAGAGTATGACTGGGAAGACGCCTGATGTCGACTTCCGTCT
    ||||||||||||||||||||||||||||||||||||||||||
KO  CTGTCTCAAGAAGATAAAGTAGAGTATGACTGGGAAGACGCC------------------

WT  TTATCACGCATGCTTGCATACCAACATGCACTTGTGTGTGTGCCCATACAAATGCATTCA
KO  ------------------------------------------------------------

WT  TACATGCAAATACAGAAACACAATATACAATATAAGGCTTTTCAAACCTTGCCTGATCTC
KO  ------------------------------------------------------------

WT  CAGAAAAACTCGGTGGCAATCAGCTAGTGACTTAGTTATTATTATTTATATGTTAGTGAA
KO  ------------------------------------------------------------

WT  TGTCCCCAGAGGACATCTGATGGGCTAGCTTCTGCTGTCAGGGCAGCATCCATAACAGGA
KO  ------------------------------------------------------------

WT  CATCCAAGAACCCCATGCAGGATCTGAGGGCCATGGTACTCTGTCGCTATCCTTCACCCC
KO  ------------------------------------------------------------

WT  TGCATGGCTTCTCACACCTCTCCCCTCCCTCTTCTGCCCAGAGCCCCCCAAAGTGAGGCT
KO  ------------------------------------------------------------

                          Exon 6
WT  GGAGGACCGAAGCACCACCTCCCTGAGTGTCACCTGGAGCATCCCGGTGTCACAGCAGAG
KO  ------------------------------------------------------------

WT  CCGTGTGTGGAAGTACGAAGTCACCTACCGCAAGAAGGTAAACTCGATGGGGGTTGGCAC
KO  ------------------------------------------------------------

WT  AGGCCATGGAGATATTCAGTGCACAGCGATCTGGAAGGGTTCCCTTCTGCGTCTGGGGAA
KO  ------------------------------------------------------------

WT  GCTGAGACCTGGGGTTTCCTAGAAGCTTATAGGGGTGACTATTTTGGGTTCTCAGAGCCA
KO  ------------------------------------------------------------

WT  GTTAGAATGAGCTCTTGGAATTAAGCTAGTCCTGGGGATGTATAGTCCCCCTCTGGCCTC
                                                    ||||||||||||
KO  ------------------------------------------------CCCTCTGGCCTC
```

Figure S1. Sequence information of the Epha2 gene in the generated Epha2 knockout mouse.
(A) Schematic diagram of gene modification in Epha2 KO mice using the CRISPR/Cas9 system. (B) Genomic sequence analysis result for Epha2 KO mice. Comparison of Epha2 genomic sequence in WT (top) and Epha2 KO (bottom) mice. The exon sequence is highlighted in blue.

Figure S2

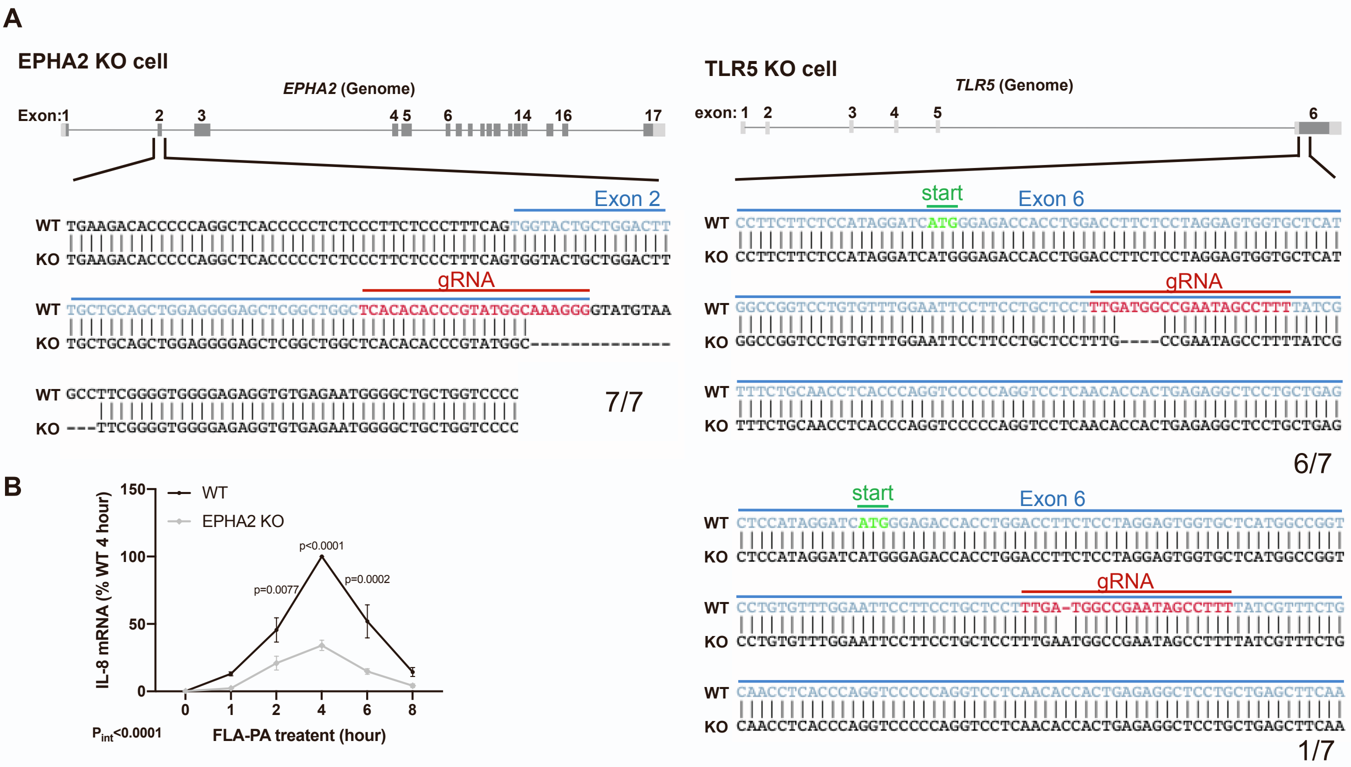

Figure S2. Analysis result of genome sequence and inflammatory response of engineered CRISPR KO cell lines.

(A) Results of genome sequencing analysis for BEAS-2B EPHA2 KO and TLR5 KO cells. The light gray regions on the genome map represent UTR in Exons. The top sequence displays the normal sequence, while the bottom sequence presents the sequence result of the KO cells. The numbers below the sequence represent the number of clones analyzed (denominator) and the frequency of the shown sequence being read (numerator).

(B) WT or EPHA2 KO BEAS-2B cells were treated with 100 ng/ml FLA-PA for 0-8 hours, and the IL-8 mRNA level was analyzed by RT-qPCR. IL-8 mRNA expression was compared between WT and EPHA2 KO cells. n=3. ±SEM, (RM two-way ANOVA, Sidak's test).

Figure S3

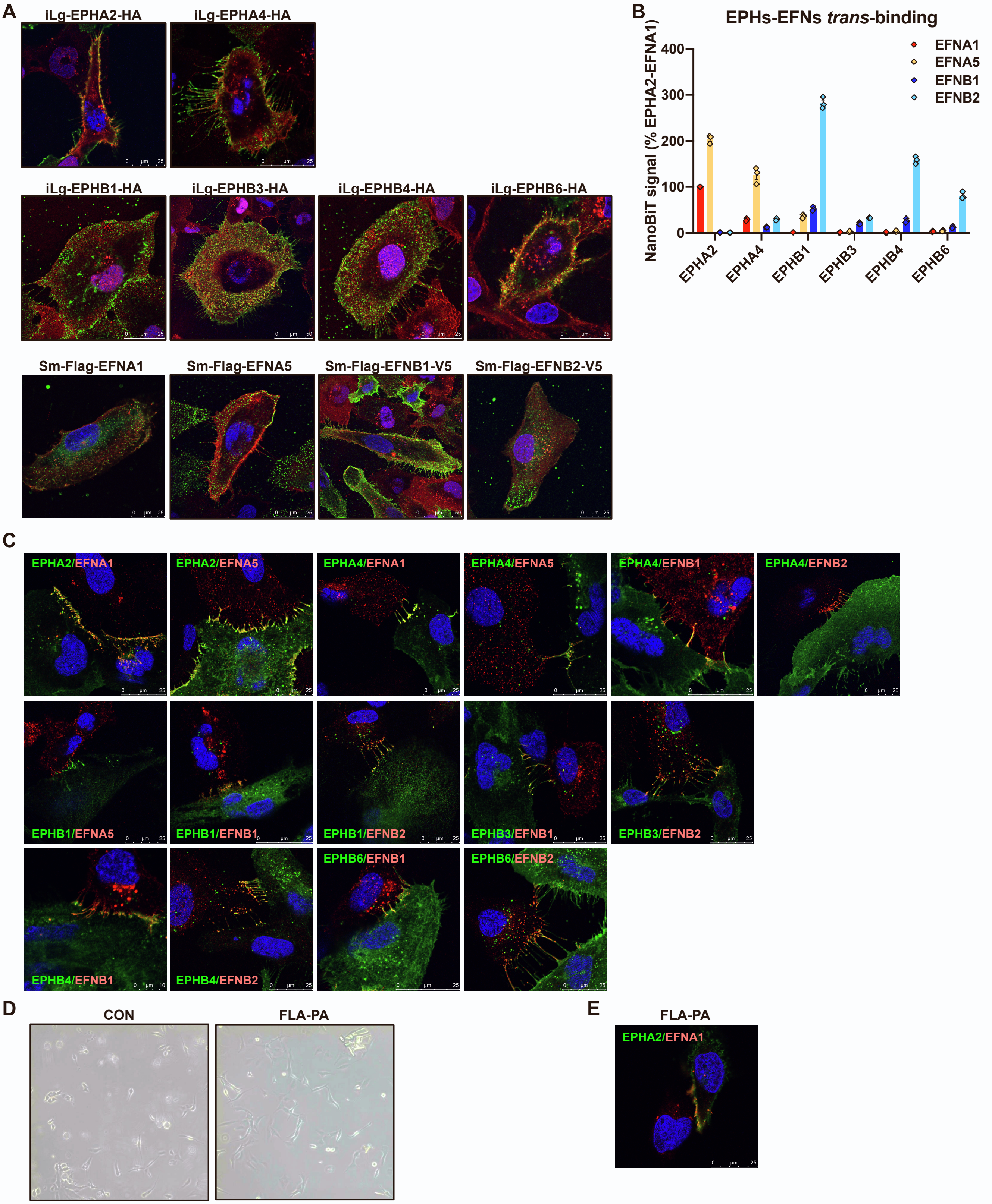

Figure S3. Localization and binding analysis of expressed iLg-EPH and Sm-Flag-EFN in BEAS-2B cells.
(A) Cellular localization of exogenously expressed iLg-EPH-HA and Sm-Flag-EFN in BEAS-2B cells. iLg-EPH-HA were induced by treating with 1 μg/ml Dox for 2 days. The membrane was permeabilized, and immunofluorescence staining was carried out using antibodies against the HA tag and Flag tag, along with Alexa Fluor 488-labeled anti-mouse IgG. The plasma membrane was stained with Alexa Fluor 594 conjugated Wheat Germ Agglutinin (red), and nuclei were stained with DAPI (blue).
(B) A bar graph displaying the same data as Figure 3G. ±SEM.
(C) BEAS-2B cells expressing iLg-EPH-HA or Sm-Flag-EFN were co-cultured in the respective patterns, and the cell-cell contact sites were observed through immunofluorescence staining, as shown in Figure 3F.
(D) Bright-field images (scale bar indicates 100 μm) or (E) immunofluorescence images of co-cultured iLg-EPHA2-HA and Sm-Flag-EFNA1 expressing BEAS-2B cells 4 hours after 100 ng/ml FLA-PA treatment.

Figure S4

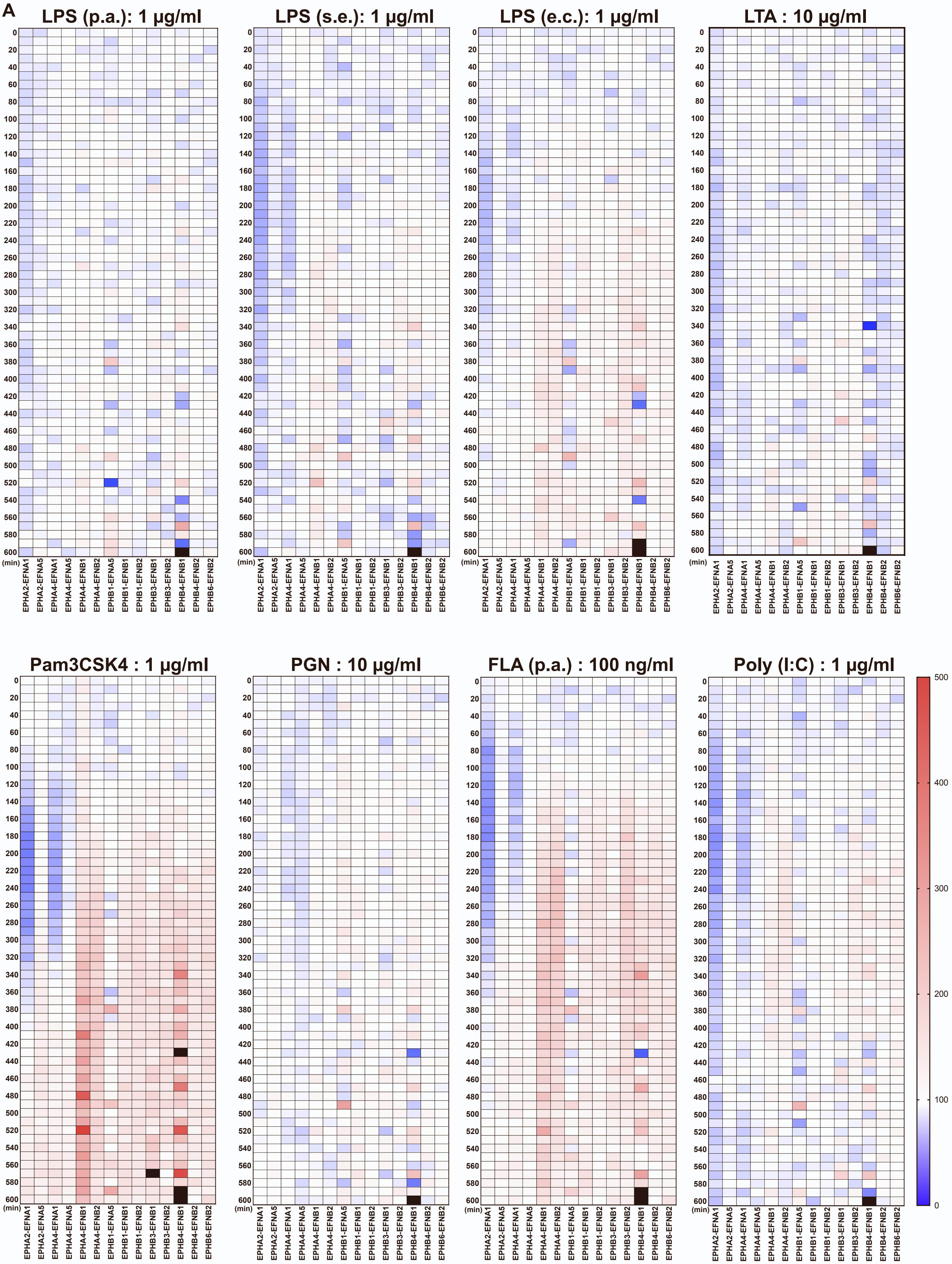

A
LPS (p.a.): 1 μg/ml
LPS (s.e.): 1 μg/ml
LPS (e.c.): 1 μg/ml
LTA : 10 μg/ml
Pam3CSK4 : 1 μg/ml
PGN : 10 μg/ml
FLA (p.a.) : 100 ng/ml
Poly (I:C) : 1 μg/ml
(min)
0
20
40
60
80
100
120
140
160
180
200
220
240
260
280
300
320
340
360
380
400
420
440
460
480
500
520
540
560
580
600
EPHA2-EFNA1
EPHA2-EFNA5
EPHA4-EFNA1
EPHA4-EFNA5
EPHA4-EFNB1
EPHA4-EFNB2
EPHB1-EFNA5
EPHB1-EFNB1
EPHB1-EFNB2
EPHB3-EFNB1
EPHB3-EFNB2
EPHB4-EFNB1
EPHB4-EFNB2
EPHB6-EFNB2
500
400
300
200
100
0

Figure S4 - continued

A

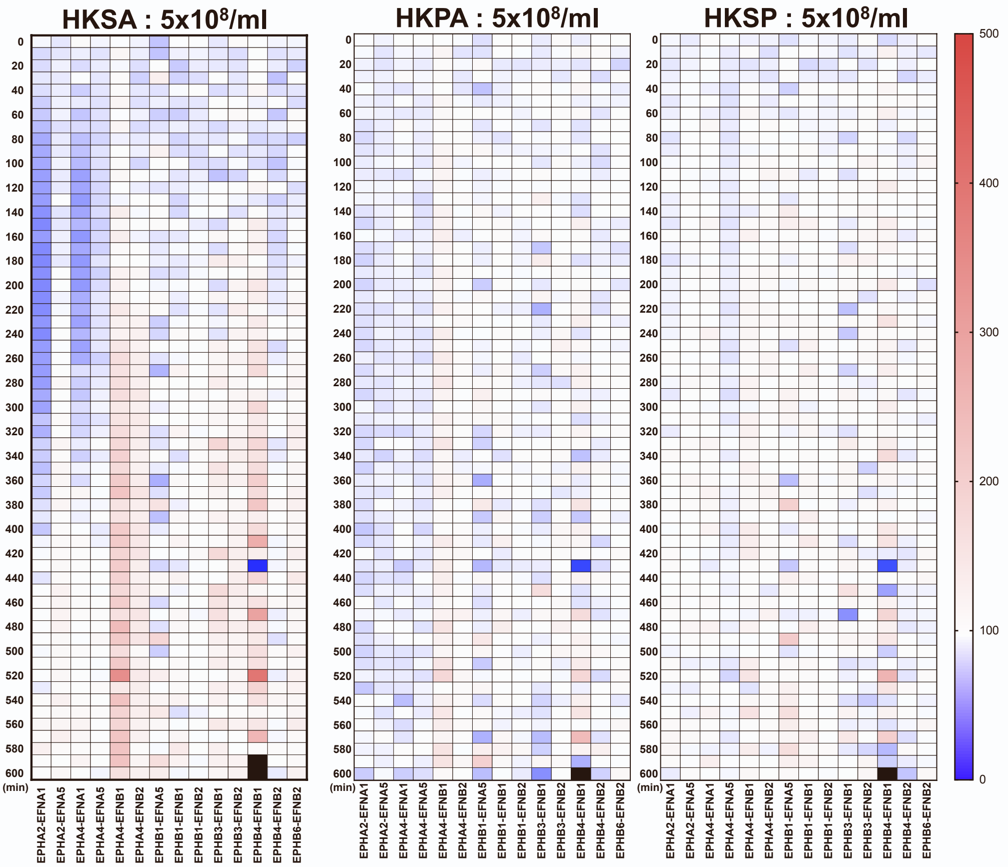

B

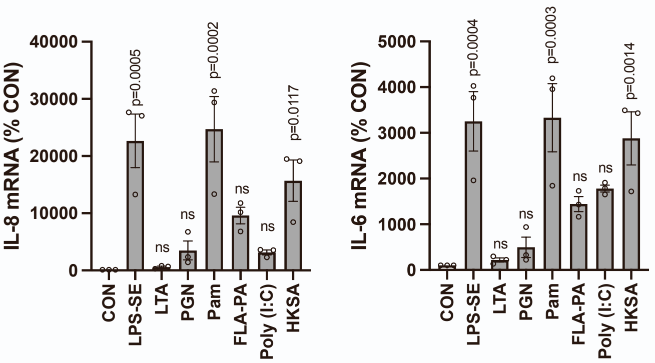

Figure S4. Results of the EPH-EFN interaction screening and evaluation of cytokine induction in response to various stimuli.

(A) The complete time course results from Figure 4A were presented in a heatmap. The heatmap illustrates the percentage of EPH-EFN interaction relative to the CON value (100%) at each time point. The color scale ranges from 0% (blue) to 100% (white), with a maximum of 500% (red). Points where the signal was not detected are labeled in black. n=3.

(B) BEAS-2B cells were treated with the indicated pathogen components for 4 hours, and IL-8 and IL-6 mRNA expression levels were quantified by RT-qPCR. The concentration of pathogen components was the same as in Figure 2. n=3. ±SEM, (Non-RM one-way ANOVA, Dunnett's test).

Figure S5

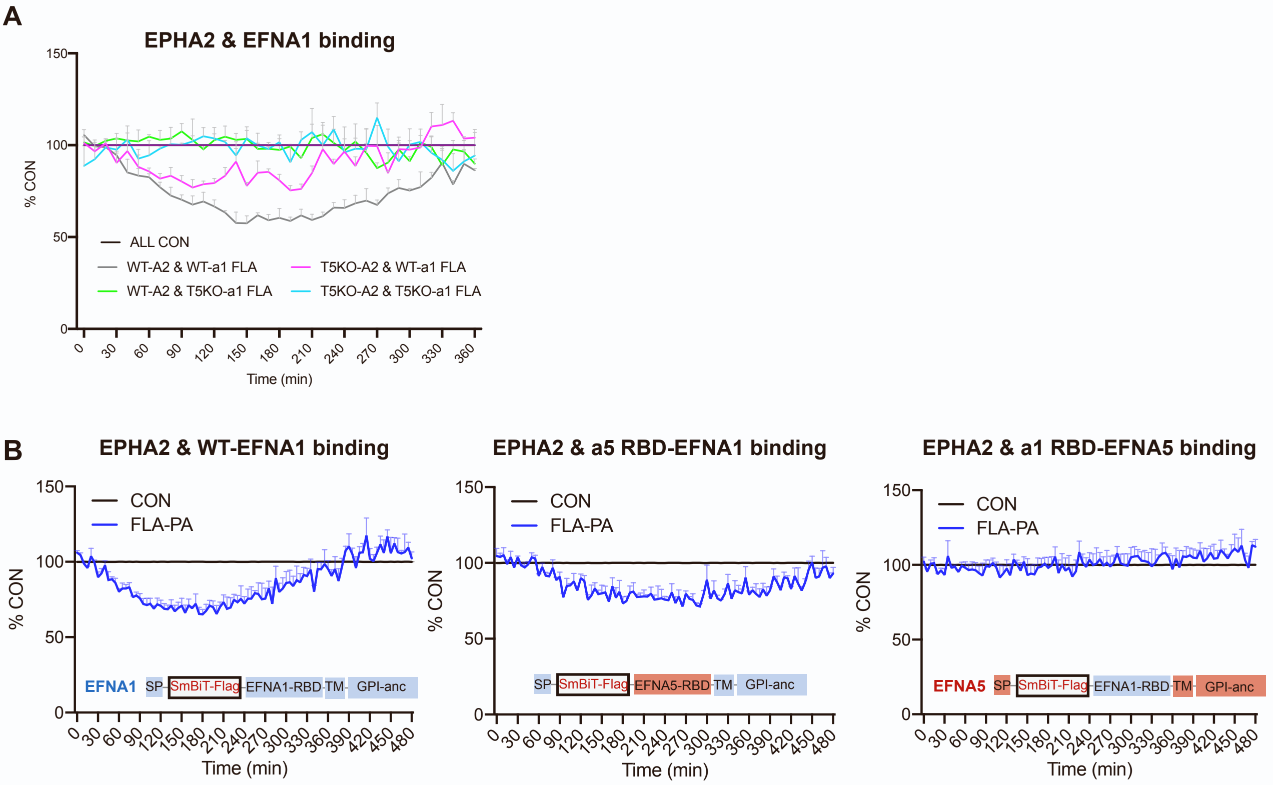

Figure S5. Time course results of EPHA2 and WT or chimera EFNA1 binding, related to Figure 5.
(A) Time course result of the Figure 5D. A2 and a1 represent iLg-EPHA2-HA- and Sm-Flag-EFNA1-expressing cells, respectively. n=3. +SEM.
(B) Time course results of the Figure 5E. n=3. ±SEM.

Figure S6

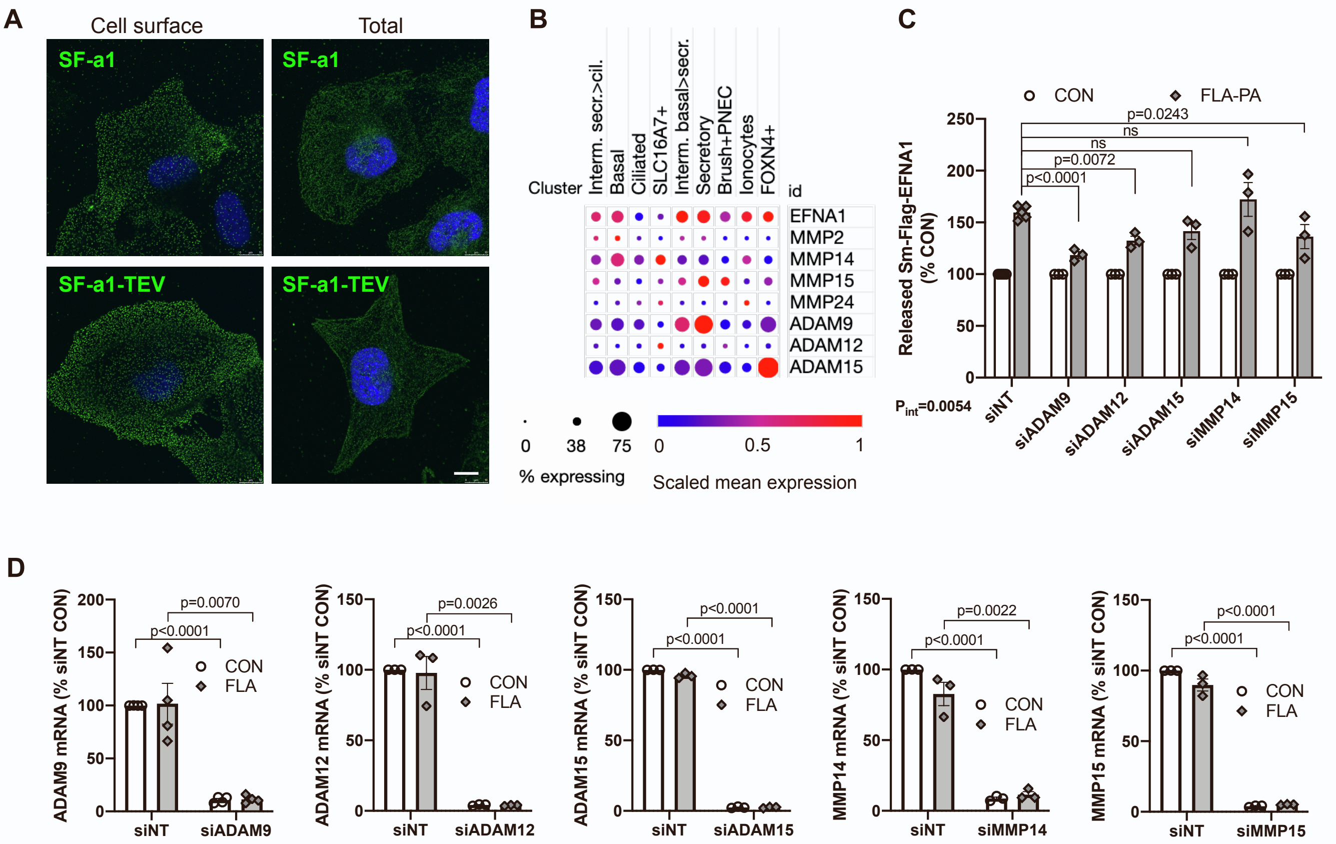

Figure S6. Analysis of the involvement of MMP/ADAM in FLA-PA-induced cleavage of EFNA1, related to Figure 6.

(A) Subcellular localization analysis of SF-a1 and SF-a1-TEV in BEAS-2B cells. Immunofluorescence staining was performed in BEAS-2B cells expressing SF-a1 or SF-a1-TEV with anti-Flag and anti-mouse-AlexaFluor 488 antibodies under non-permeabilized (Cell surface) and permeabilized (Total) conditions. The nucleus was stained with DAPI. The scale bar indicates 10 μm.

(B) Comparison of EFNA1 and MMPs/ADAMs expression in airway epithelial cell populations based on single-cell RNA-seq results from Lindsey W. Plasschaert et al.'s paper 35 (Single Cell Portal, #SRR5881096).

(C) The Figure 6K results were re-analyzed by normalizing all values to the CON of each group. siNT: n=5, Other groups: n=3. ±SEM, (RM two-way ANOVA, Dunnett's test).

(D) Analysis of ADAMs and MMPs siRNA KD efficiency in the Figure 7E experiment by RT-qPCR. n=3. ±SEM, (Unpaired multiple t-test, Bonferroni-Dunn test).

Figure S7

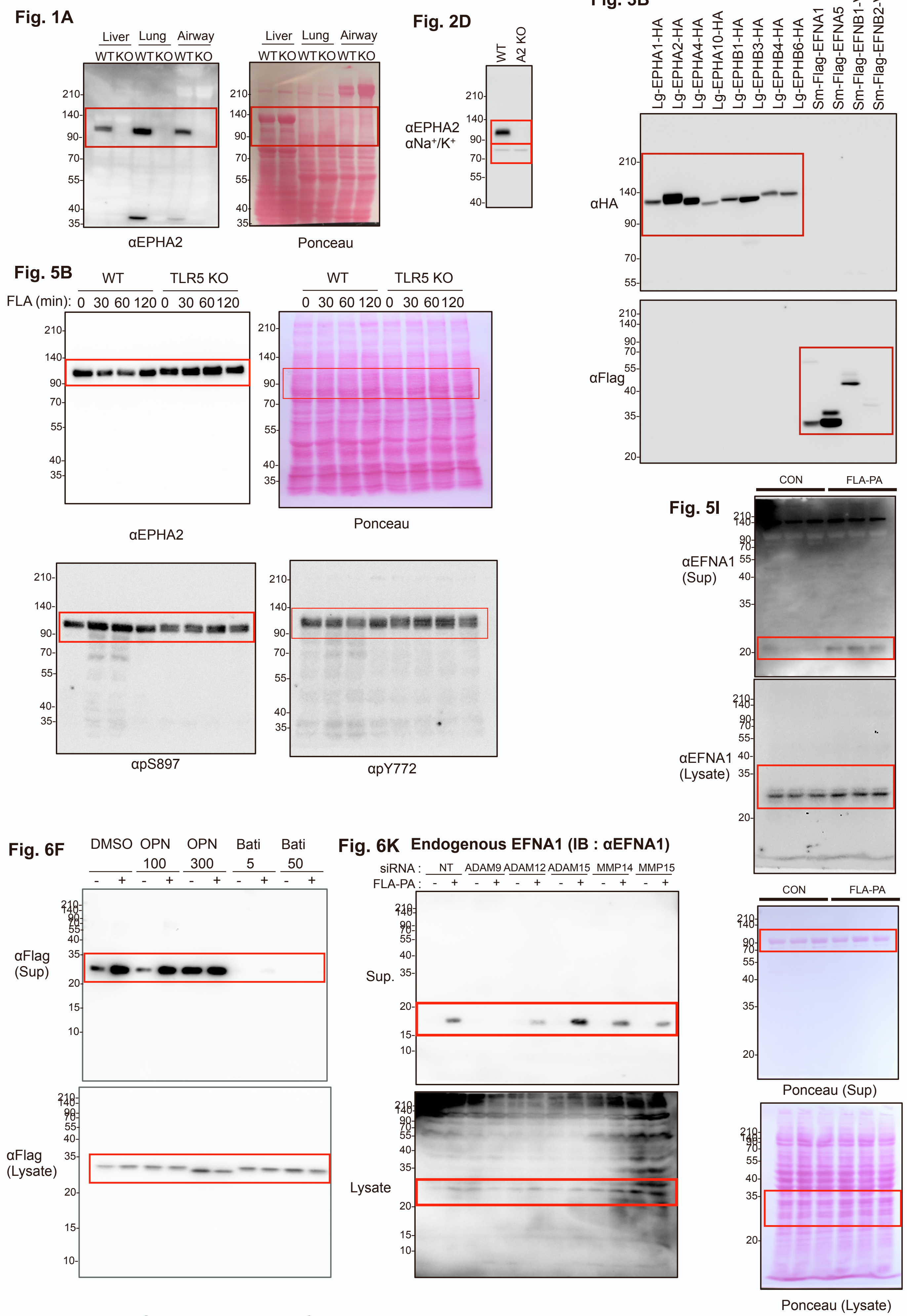

Figure S7. Full gel images of immuno-blot analysis.

Figure S8

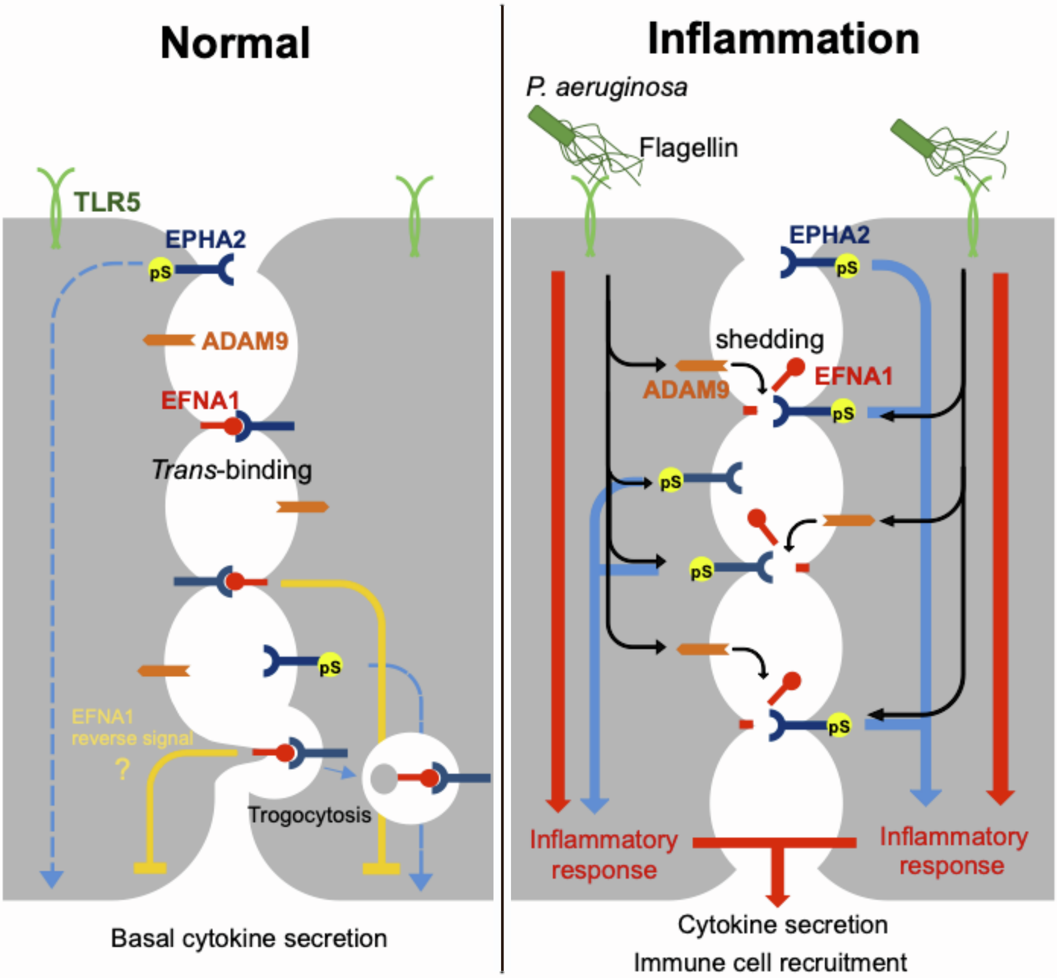

Figure S8. Graphical overview of this research.

In the absence of inflammatory stimulation, EPHA2-EFNA1 trans-binding is maintained between adjacent AECs. In the ligand-unbound state, EPHA2 S897 phosphorylation occurs, which is presumed to be involved in the minimal cytokine secretion observed in steady-state conditions. The reverse signaling of EFNA1 through receptor binding may function suppressively in the cell's inflammatory response (indicated by the yellow inhibitory arrow). When inflammation is induced by pathogens or their components such as *P. aeruginosa* or flagellin, the main inflammatory response signal (i.e. NF-κB signal) occurs through PRRs like TLRs (red arrow). In parallel, FLA-PA-induced TLR5 signaling leading to EPHA2 S897 phosphorylation and rapid shedding of EFNA1 mediated by ADAM9 (black arrow). EFNA1 shedding induces transient dissociation of EPHA2-EFNA1 trans-binding. EFNA1 shedding in the absence of inflammatory stimulation has been shown to induce a 3-5 folds increase in IL-8 expression, suggesting that this inflammation amplification pathway (blue arrow) determines the inflammatory responsiveness of AECs in coordination with the main inflammatory stream. The silencing of EPHA2, EFNA1, or ADAM9 suppressed the inflammatory response in AECs, indicating that the transient dissociation of EPHA2-EFNA1 mediated by ADAM9 during inflammation assists in appropriate inflammatory response that leads to pathogen clearance.
